# Efficacy and safety of exercise rehabilitation across the valve intervention pathway: A systematic review and meta-analysis

**DOI:** 10.3389/fcvm.2026.2004830

**Published:** 2026-10-29

**Authors:** Shanshan Lin, Haitao Wang, Ying Zhu, Dian Zhao, Tao Yu, Keli Huang, Xinlei He, Qin Jiang

**Affiliations:** 1 Department of Cardiac Surgery, Sichuan Provincial People’s Hospital, Affiliated Hospital of University of Electronic Science and Technology, Chengdu, China; 2 Department of Urology, Sichuan Academy of Medical Sciences & Sichuan Provincial People’s Hospital, School of Medicine, University of Electronic Science and Technology of China, Chengdu, China

**Keywords:** cardiac rehabilitation, exercise capacity, exercise training, meta-analysis, transcatheter aortic valve replacement, valve surgery, valvular heart disease

## Abstract

**Background:**

The benefits and safety of exercise-based rehabilitation across the valve intervention pathway remain uncertain. We assessed its effects on exercise capacity, cardiac function, postoperative recovery, and reported adverse events.

**Methods:**

We searched eight English and Chinese databases for randomised controlled trials of rehabilitation initiated before, around, or after surgical or transcatheter valve intervention. Outcomes were 6-min walk distance (6MWD), peak oxygen uptake (peak VO_2_), left ventricular ejection fraction (LVEF), postoperative length of stay, reported adverse events, and pulmonary complications. Principal analyses used random-effects models. We assessed outcome-specific risk of bias with RoB 2 and evidence certainty with GRADE, and performed subgroup interaction and sensitivity analyses. PROSPERO registration: CRD420261509016.

**Results:**

Twenty-nine trials were included. Rehabilitation increased 6MWD (19 trials; mean difference [MD] 40.95 m, 95% CI 27.10 to 54.81), peak VO_2_ [eight trials; standardised mean difference (SMD) 0.32, 0.07 to 0.57], and LVEF (14 trials; MD 4.41 percentage points, 2.26 to 6.56), and shortened postoperative hospital stay (16 trials; MD −1.77 days, −2.41 to −1.13). Reported adverse events were less frequent [14 estimable trials; risk ratio (RR) 0.53, 0.33 to 0.85], as were pulmonary complications (seven trials; RR 0.62, 0.50 to 0.77). Heterogeneity was substantial for 6MWD, LVEF, and hospital stay. Prediction intervals crossed the null for all outcomes except pulmonary complications. The peak VO_2_ result was no longer statistically significant after excluding high-risk results. Evidence certainty was low for pulmonary complications and very low for the other outcomes.

**Conclusion:**

Exercise-based rehabilitation may improve functional and postoperative outcomes around valve intervention, but the magnitude and applicability of benefits remain uncertain. Diverse event definitions prevent the pooled adverse-event result from establishing exercise-specific safety. Larger trials with standardised interventions, outcome assessment, and longer follow-up are needed.

## Introduction

Valvular heart disease (VHD) is a major cardiovascular condition that alters cardiac structure and blood flow. Its global burden is changing substantially because of population ageing and broader epidemiological shifts. According to estimates from the Global Burden of Disease Study 2021, approximately 54.8 million people worldwide had rheumatic heart disease, while calcific aortic valve disease and degenerative mitral valve disease affected an estimated 13.3 million and 15.5 million people, respectively (1). Surgical valve repair or replacement and transcatheter procedures are key treatments for clinically significant valve disease, but correction of valve dysfunction does not necessarily restore physical function (2). Open-heart surgery can cause chest-wall injury, inflammation related to cardiopulmonary bypass, reduced respiratory function, and prolonged immobility, thereby increasing the risks of pulmonary complications and muscle loss (3). Patients undergoing transcatheter aortic valve replacement (TAVR) are often older and may also have frailty, reduced muscle mass, and multiple coexisting conditions. Thus, even after valve obstruction has been relieved, impaired exercise capacity and limitations in daily activities may persist (2). Differences in operative burden, baseline functional capacity, and frailty may therefore influence rehabilitation needs and responses in surgical and TAVR populations (4). Post-procedural care should therefore extend beyond anatomical correction to prioritise recovery of physical function and daily independence.

Exercise-based rehabilitation aims to support functional recovery and may be initiated before, around, or after a valve procedure. Programmes vary and may include aerobic and resistance training, inspiratory muscle training, breathing exercises, early mobilisation, or combinations of these components (2, 3, 5). Aerobic and resistance training target exercise tolerance and peripheral muscle function, whereas respiratory training and early mobilisation aim to support pulmonary recovery and limit the consequences of inactivity (2, 3, 5, 6). Some programmes also incorporate psychological support, nutritional care, and health education, which may influence adherence and recovery (7). However, these programmes differ in their timing, intensity, duration, supervision, and accompanying care. Control interventions also range from usual care without structured exercise to routine rehabilitation, basic exercise, or sham training. Consequently, the trials estimate the effects of different additional rehabilitation components rather than one uniform intervention. Rehabilitation outcomes are commonly assessed using the 6-min walk distance (6MWD), peak oxygen uptake (peak VO_2_), left ventricular ejection fraction (LVEF), postoperative length of stay (LOS), and incidence of adverse events (8–11). Reported adverse events may include exercise-related symptoms, general postoperative complications, or other clinical events; these outcomes should be distinguished when assessing the safety of rehabilitation itself.

Evidence from randomised trials and systematic reviews remains mixed. In the SPORT:TAVI pilot trial, 8 weeks of combined aerobic and resistance training improved peak VO_2_ compared with usual care (10). The CopenHeartVR trial also reported short-term improvements in physical capacity following a programme combining exercise and psychoeducation (12). Evidence also extends to preoperative rehabilitation, including the TIME trial and the inspiratory muscle training trial by Yu and colleagues, which evaluated postoperative pulmonary outcomes (3, 11). However, larger evidence reviews have been less conclusive. A 2021 Cochrane review of six randomised trials involving 364 participants concluded that effects on mortality, hospitalisation, and health-related quality of life remained uncertain because the evidence was of very low certainty (13). A 2026 meta-analysis restricted to TAVR found no statistically significant improvements in 6MWD, peak VO_2_, or LVEF (8). Other meta-analyses have reported benefits for selected functional and postoperative outcomes (9, 14). These differing findings may reflect variation in study populations, rehabilitation components, comparator care, and outcome timing, together with small samples and methodological limitations. An updated synthesis should therefore consider these clinical differences and the certainty of the evidence when interpreting pooled effects.

Given the fragmented evidence, we systematically evaluated the effects of exercise-based rehabilitation initiated before, around, or after surgical or transcatheter intervention for VHD. The main outcomes were 6-min walk distance and peak oxygen uptake, which measure exercise capacity and cardiorespiratory fitness. We also assessed left ventricular ejection fraction, postoperative length of stay, reported adverse events, and postoperative pulmonary complications, while distinguishing general postoperative morbidity from harms attributable to exercise. We examined differences by procedure type, intervention duration, and rehabilitation modality using formal tests of subgroup differences. Robustness was assessed through sensitivity analyses addressing individual studies, outcome-specific risk of bias, analytical assumptions, and outcome timing where sufficient data were available. By combining updated trial evidence with explicit assessment of clinical heterogeneity and outcome-specific certainty, this review aimed to clarify the potential benefits and limitations of rehabilitation across the valve intervention pathway.

## Methods

This systematic review and meta-analysis was reported with reference to the PRISMA 2020 statement, with the checklist provided in Supplementary Appendix 1 (15). The research question was defined using the PICOS framework, covering participants, interventions, comparators, outcomes, and study design. The protocol was registered in PROSPERO (CRD420261509016).

### Data sources and search strategy

We searched PubMed, Web of Science, Embase, the Cochrane Library, CNKI, WanFang Data, VIP, and SinoMed from their inception to Sept 10, 2026. We included randomised controlled trials that assessed the benefits and safety of exercise-based rehabilitation initiated before, around, or after surgical or transcatheter treatment for valvular heart disease. The search combined database-specific subject headings and keywords related to valvular heart disease, valve repair or replacement, transcatheter valve procedures, exercise-based rehabilitation, aerobic and strength training, breathing exercises, early mobilisation, and randomised trials. Equivalent Chinese terms were used in Chinese databases. The full search strategies are provided in Appendix 2.

### Inclusion and exclusion criteria

Studies were eligible if they met the following criteria:

- Study design: Parallel-group randomised controlled trials using individual or cluster randomisation.
- Participants: Adults aged 18 years or older with valvular heart disease who underwent surgical valve repair or replacement, or a transcatheter valve procedure, including surgical aortic valve replacement, mitral valve repair or replacement, and TAVR/TAVI. Studies of preoperative or perioperative rehabilitation were eligible if they reported postoperative outcomes.
- Interventions: Exercise-based rehabilitation, including aerobic exercise, strength training, combined exercise, inspiratory muscle training, breathing exercises, early mobilisation, home-based exercise, or comprehensive cardiac rehabilitation. Programmes could also include education, psychological support, nutritional care, and management of cardiovascular risk factors, provided that exercise was a main component.
- Comparators: Usual care or treatment, standard rehabilitation, basic exercise advice, health education, sham training, or no structured exercise programme. Active rehabilitation comparators were eligible when the intervention provided an additional exercise or rehabilitation component.
- Outcomes: At least one relevant measure of benefit or safety, including 6-min walk distance, peak oxygen uptake, left ventricular ejection fraction, postoperative length of stay, reported adverse events, or pulmonary complications. Studies also had to provide sufficient data to calculate the treatment effect.

We excluded non-randomised, animal, and laboratory studies. Studies were also excluded if participants had not undergone surgical or transcatheter valve treatment; the intervention did not include exercise-based rehabilitation; or both groups received the same rehabilitation components without a distinct intervention contrast. We further excluded studies that did not report relevant outcomes or provide sufficient numerical data. For duplicate reports or studies with overlapping participants, we selected the publication with the largest sample, most complete follow-up, or most comprehensive outcome data.

### Study selection and data extraction

All retrieved records were imported into reference-management software and duplicates were removed. Two reviewers independently screened titles and abstracts, assessed potentially eligible full-text articles against the prespecified criteria, and resolved disagreements through discussion or consultation with a third reviewer. The selection process was summarised in a PRISMA flow diagram. The available full-text exclusion register and report-specific reasons are provided in Supplementary Table S2. Using a standardised form, two reviewers independently extracted the first author, publication year, country, sample size, mean age, body-mass index, procedure type, rehabilitation programme, exercise frequency and intensity, intervention duration, comparator, and follow-up period. Where available, we also extracted valve disease, sex, baseline NYHA class, LVEF, 6MWD, peak VO_2_, and frailty. Intervention details included session duration, supervision, initiation relative to the valve procedure, and components received by the control group (Table 1; Supplementary Tables S3–S7). For continuous outcomes—6-min walk distance, peak oxygen uptake, left ventricular ejection fraction, and postoperative length of stay—we recorded the mean, standard deviation, and sample size for each group. For binary outcomes—reported adverse events and postoperative pulmonary complications—we recorded the number of events and participants in each group.

**Table 1:** Baseline characteristics of included studies.

Study	Country	Procedure	Age (T/C), years	BMI (T/C), kg/m^2^	Sample size (T/C)	Intervention	Control	Rehabilitation phase	Start time	Intervention duration	Follow-up
Pressler 2016 (10)	Germany	TAVR/TAVI	81 ± 7/81 ± 5	26.9 ± 3.1/26.9 ± 3.7	15/15	Aerobic + resistance training	Usual care	Post-op	∼3 mo post-TAVR	8 wk	End of training
Cargnin 2019 (16)	Brazil	Surgery	62.8 ± 8.8/60.1 ± 12.5	26.7 ± 1.7/28.1 ± 1.4	13/12	IMT	Sham IMT	Post-op	POD 3	4 wk	End of training
Nilsson 2018 (17)	Sweden	Surgery	58.5 (39–75)/65.5 (60–71)	NR	6/6	Aerobic training	Exercise advice	Post-op	5–6 wk post-procedure	12 wk	End of training; 1 yr later
Rogers 2018 (18)	United Kingdom	TAVR/TAVI	82.92 ± 6.0/81.21 ± 3.6	27.24 ± 3.6/28.13 ± 4.9	13/14	Aerobic + resistance CR	Usual care	Post-op	1 mo post-TAVR	6 weekly sessions	3 and 6 mo post-randomization
Sire 1987 (19)	Norway	Surgery	45.5 ± 11.7/45.5 ± 12.2	NR	21/23	Exercise + vocational support	No structured exercise	Post-op	2 mo post-procedure	4 wk	6 and 12 mo post-op
Cao 2013 (20)	China	Surgery	48.5 ± 8.4/49.2 ± 13.5	NR	50/47	Mobilization + education	Usual care	Post-op	Post-op inpatient period	Inpatient; duration NR	Pre-discharge
Li 2020 (21)	China	Surgery	56.34 ± 2.52/56.15 ± 2.62	23.97 ± 2.42/24.21 ± 2.52	40/40	Exercise + motivational interviewing	Usual care	Pre-op	7 d pre-op	7 d	POD 7; 1 mo post-op
Lin 2004 (22)	China	Surgery	32.8 ± 12.1/29.8 ± 9.4	NR	55/49	Breathing + exercise	Usual care	Peri-op	1 wk pre-op	Pre-op/inpatient + 3 mo home training	Post-op; 3 mo
Sun 2021 (23)	China	Surgery	69.1 ± 2.7/70.1 ± 3.8	24.1 ± 2.7/25.1 ± 2.3	100/100	Individualized exercise	Usual care	Pre-op	Pre-op admission	1 wk	After care; timing unclear
Zou 2020 (24)	China	Surgery	53.21 ± 2.68/53.78 ± 2.91	NR	40/40	Exercise + nursing support	Usual care	Post-op	POD 2–3	Inpatient; duration NR	After care; timing NR
Tao 2020 (25)	China	Surgery	82.32 ± 1.26/82.34 ± 1.14	NR	35/35	Early mobilization	Usual care	Post-op	During post-op ventilation	Until discharge	Pre-discharge
Tamulevičiūtė-Prascienė 2021 (26)	Lithuania	Surgery	75.9 ± 6.6/76.4 ± 6.6	27.9 ± 3.9/27.6 ± 4.9	60/56	Resistance + balance training + CR	Standard CR	Post-op	∼2 wk post-procedure	20 d CR + 12 wk advice/contact	End of CR; 3 mo later
Weber 2021 (27)	Germany	TAVR/TAVI	82.2 ± 5.8/81.7 ± 5.0	26.3 ± 4.7/27.4 ± 4.5	58/50	Prehabilitation + physiotherapy	Standard physiotherapy	Peri-op	≥2 wk pre-TAVR	Pre-TAVR to discharge	Inpatient; 30 and 90 d
Xue 2022 (5)	China	Surgery	58.20 ± 5.27/57.98 ± 4.18	23.09 ± 2.23/23.28 ± 2.59	44/43	Mobilization + cycling	Usual care	Post-op	POD 1	Until discharge	Discharge; 6 mo later
Xu 2022 (28)	China	TAVR/TAVI	72.01 ± 5.56/72.45 ± 7.68	22.72 ± 3.72/22.65 ± 3.17	48/48	IMT + CR	Sham IMT + CR	Post-op	Post-TAVR; CR from POD 2	Inpatient	Discharge; 1 and 3 mo later
Hu (ENERGY) 2023 (29)	China	TAVR/TAVI	70.36 ± 6.93/70.94 ± 6.35	23.96 ± 3.78/24.29 ± 3.31	33/33	Aerobic training	Exercise advice	Post-op	≥1 mo post-TAVR	3 mo	3 mo post-randomization
Vitez 2023 (30)	Slovenia	TAVR/TAVI	81 ± 5/82 ± 4	30.9 ± 4.9/26.3 ± 5.2	20/19	Multicomponent exercise	Home exercise	Post-op	3–6 mo post-TAVR	8–12 wk	End of training
Song 2024 (31)	China	TAVR/TAVI	76.8 ± 3.4/75.6 ± 3.5	23.9 ± 3.9/23.8 ± 2.3	30/30	Aerobic training	Non-exercise CR	Post-op	Post-TAVR; interval NR	12 wk	End of training
Shi 2025 (32)	United States	TAVR/TAVI	85.1 ± 9.1/84.9 ± 5.1	27.5 ± 6.4/27.9 ± 4.8	15/18	Home exercise + CBI	Education + walking advice	Post-op	≤14 d post-discharge	8 wk	4 and 8 wk
Gao 2025 (33)	China	Surgery	56.27 ± 6.63/54.91 ± 9.87	22.06 ± 3.30/23.18 ± 3.04	95/95	ERAS rehabilitation	Standard rehabilitation	Post-op	POD 1 (added programme)	Inpatient + follow-up support	Inpatient; up to 1 mo
Liu 2025 (34)	China	Surgery	52 ± 11.6/51 ± 12.3	23.4 ± 3.5/23.3 ± 3.5	492/486	IMT + breathing + mobilization	Unsupervised respiratory care	Peri-op	Pre-op admission	Pre-op to discharge	Inpatient; 3 mo (mortality)
Li 2023 (35)	China	Surgery	59.30 ± 3.27/58.66 ± 3.49	NR	46/46	Breathing + exercise	Usual care + mobilization	Post-op	During post-op ventilation	3 mo	End of intervention
Gao 2024 (36)	China	Surgery	NR	NR	40/40	Video-guided exercise + CBT	Early mobilization	Post-op	Post-op; day NR	NR	Pre-discharge; 3 mo
Zhang 2026 (37)	China	Surgery	48.73 ± 3.97/48.62 ± 3.58	NR	40/40	Mobilization + breathing	Usual care	Post-op	≤12 h post-op	Early inpatient; duration NR	After care; timing NR
Sibilitz 2016 (12)	Denmark	Surgery	62.0 ± 11.5/61.0 ± 9.9	26.2 ± 4.2/26.1 ± 3.9	72/75	Exercise-based CR + psychoeducation	Usual care	Post-op	1 mo post-op (exercise)	12 wk exercise + 5 monthly consultations	4 mo (physical outcomes); 6 mo (PROs)
Luo 2023 (11)	China	Surgery/TAVR	63.48 ± 10.90/63.34 ± 9.61	23.98 ± 3.67/23.35 ± 3.28	82/83	IMT + walking + education	Usual care	Pre-op	3 d pre-procedure	3 d	14 d (PPCs); 3 mo (QoL)
Yu 2025 (3)	China	Surgery	54 ± 9/55 ± 9	24.5 ± 3.8/24.6 ± 4.1	76/76	IMT	Sham IMT	Pre-op	3 d pre-procedure	3 d; extended if delayed	Pre-op; discharge; 1 mo later
Ahmad 2024 (38)	Egypt	Surgery	33.44 ± 5.77/33.20 ± 6.29	28.11 ± 6.41/29.83 ± 8.36	19/19	Bedside cycling + CR	Standard CR	Post-op	POD 1	Until discharge	Discharge; 1 mo later
Atef 2026 (39)	Egypt	Surgery	26.85 ± 3.66/28.30 ± 3.20	26.27 ± 3.98/26.31 ± 5.07	20/20	Aerobic + resistance training	Usual care	Post-op	≤48 h post-op	4 wk	End of training

Values separated by a slash are presented for the intervention and control groups, respectively. Age and BMI are reported as mean ± SD, except for age in Nilsson 2018, which is presented as median (minimum–maximum). Sample sizes refer to the groups listed in this table; outcome-specific analysed sample sizes may differ. Surgery denotes surgical valve repair or replacement; Surgery/TAVR denotes inclusion of both procedure types. Follow-up schedules are reported as described in the individual studies and do not necessarily represent the assessments selected for meta-analysis (Supplementary Table S8). Duration subgroup classification is based on the structured exercise course rather than subsequent advice, contact, or outcome follow-up (Supplementary Table S12).

T, intervention group; C, control group; BMI, body mass index; SD, standard deviation; TAVR, transcatheter aortic valve replacement; TAVI, transcatheter aortic valve implantation; CR, cardiac rehabilitation; IMT, inspiratory muscle training; CBI, cognitive behavioural intervention; CBT, cognitive behavioural therapy; ERAS, enhanced recovery after surgery; POD, postoperative day; PPCs, postoperative pulmonary complications; PROs, patient-reported outcomes; QoL, quality of life; NR, not reported; Pre-op, preoperative; Peri-op, perioperative; Post-op, postoperative; h, hours; d, days; wk, weeks; mo, months; yr, years. ENERGY denotes the trial name;∼indicates approximately.

One selected assessment per trial and outcome was included in the principal analysis, with the selected functional endpoints documented in Supplementary Table S8. When results were reported only as figures, medians, interquartile ranges, or ranges, means and standard deviations were estimated using established statistical methods; data extracted from figures were checked independently. During revision, the selected functional assessments were mapped to ≤30, 31–90, or >90 days after the valve procedure for timing-restricted sensitivity analyses, treating one month as 30 days. Assessments with unclear timing, intervals crossing window boundaries, or discharge without a reported postoperative day remained in the principal analysis but were excluded from timing-restricted analyses. The selected endpoint values were not changed. Hospital stay referred to the index admission. Any disagreements about data extraction were resolved through discussion or consultation with a third reviewer. Study-level definitions and observation periods for adverse events and pulmonary complications are provided in Supplementary Tables S9, S10. Events explicitly attributed to exercise were distinguished from general postoperative complications and broader clinical events when interpreting safety. The pooled adverse-event outcome was therefore not assumed to represent a uniform measure of exercise-related harm.

### Risk-of-bias assessment

Two reviewers independently assessed each selected study–outcome result using the Cochrane Risk of Bias 2 (RoB 2) tool. Assessments targeted the effect of assignment to intervention and concerned the specific outcome and assessment time included in the synthesis. The assessment covered five areas: randomisation, deviations from intended interventions, missing outcome data, outcome measurement, and selection of the reported result. Each area and the overall result were rated as low risk, some concerns, or high risk. The overall risk was rated as low when all five areas were at low risk, and as having some concerns when at least one area raised concerns but none was at high risk. Results were rated as high risk when at least one area was at high risk or concerns across several areas substantially reduced confidence in the findings. Disagreements were resolved through discussion or consultation with a third reviewer. Outcome-specific judgments and supporting reasons are presented in Supplementary Table S11.

### Certainty of evidence assessment

We assessed the certainty of evidence for each outcome using the GRADE approach. Two reviewers performed the assessments independently and resolved disagreements through discussion or consultation with a third reviewer. Evidence from randomised trials was initially considered high certainty but could be downgraded because of risk of bias, inconsistent findings, indirect evidence, imprecise estimates, or publication bias. The final certainty was rated as high, moderate, low, or very low. For outcomes with marked variation between studies, small sample sizes, wide confidence intervals, or serious risk of bias, downgrading decisions also considered the sensitivity and subgroup analyses. Indirectness was assessed by considering differences in populations, procedures, rehabilitation timing, intervention components, and comparators. Assessment of publication bias considered funnel-plot asymmetry and the limited ability to assess missing evidence when few studies were available. Outcome-specific certainty ratings and reasons for downgrading are presented in Supplementary Table S16.

### Statistical analysis

Meta-analyses were performed using Review Manager version 5.4.1. Given the clinical diversity between trials, all principal analyses used DerSimonian–Laird random-effects models with Wald 95% confidence intervals (CIs), irrespective of I^2^. Continuous outcomes were pooled using inverse-variance methods. Mean differences (MDs) were used for 6-min walk distance (metres), left ventricular ejection fraction (percentage points), and postoperative length of stay (days). Peak oxygen uptake was expressed as a standardised mean difference (SMD; Hedges' g) to accommodate differing reporting scales. Reported adverse events and postoperative pulmonary complications were analysed using the Mantel–Haenszel random-effects risk ratio (RR) option. RevMan's default continuity correction was applied where required. Studies with no events in either group did not contribute to RR estimates or subgroup-difference tests. Heterogeneity was assessed using Cochran's Q, I^2^, and *τ*^2^. Approximate 95% prediction intervals were calculated separately for the principal outcomes (Supplementary Table S13).

Subgroup analyses examined procedure type (Surgery vs. TAVR/TAVI), structured intervention duration (≤8 vs. >8 weeks), and rehabilitation modality (aerobic training, combined exercise, respiratory training/inspiratory muscle training, or early mobilisation/multimodal rehabilitation). Duration referred to the structured intervention course rather than the total follow-up period. Procedure-specific comparisons included estimates attributable to a single procedure type, and studies with unclear duration were excluded only from the duration comparison. Study-level classifications and reasons for omission from individual comparisons are provided in Supplementary Table S12.

Random-effects subgroup analyses used subgroup-specific heterogeneity estimates. Formal *χ*^2^ tests of subgroup differences were performed, and interpretation was based on these interaction tests rather than comparisons of within-subgroup statistical significance. Tests involving fewer than three estimable studies in any contributing subgroup were treated as exploratory. All tests were two-sided, with *P* < 0·05 as the nominal significance threshold. Subgroup *P* values were not adjusted for multiple comparisons (Supplementary Table S14).

Sensitivity analyses repeated the principal analyses using fixed-effect models, omitted each study in turn, and excluded results rated at high risk of bias for the specific outcome being analysed. A trial was not excluded from every outcome solely because one result was rated high risk. Additional analyses restricted functional outcomes to the defined postoperative time windows when at least three studies were available and examined the influence of uncertainty in trial design. These additional analyses were exploratory; timing-restricted estimates were not interpreted as formal tests of differences between follow-up windows. Detailed methods and results are presented in Supplementary Table S15. Funnel plots were inspected for possible publication bias and small-study effects. When fewer than ten studies were available, these plots were treated as exploratory and were not used to draw firm conclusions.

## Results

### Literature screening and included studies

The search identified 412 records: 51 from PubMed, 53 from Embase, 63 from the Cochrane Library, 49 from Web of Science, 48 from CNKI, 35 from VIP, 53 from WanFang Data, and 60 from SinoMed. No additional records were found through other sources. After removing 214 duplicates, we screened the titles and abstracts of 198 records and excluded 99. We then reviewed 99 full-text articles, of which 70 were excluded: 49 other non-randomised studies, 10 uncontrolled single-arm studies, five surveys or quality-improvement studies, five case reports, and one descriptive report without outcome data. The final analysis included 29 randomised controlled trials (3, 5, 10–12, 16–39). The study-selection process is shown in Figure 1.

**Figure 1:**
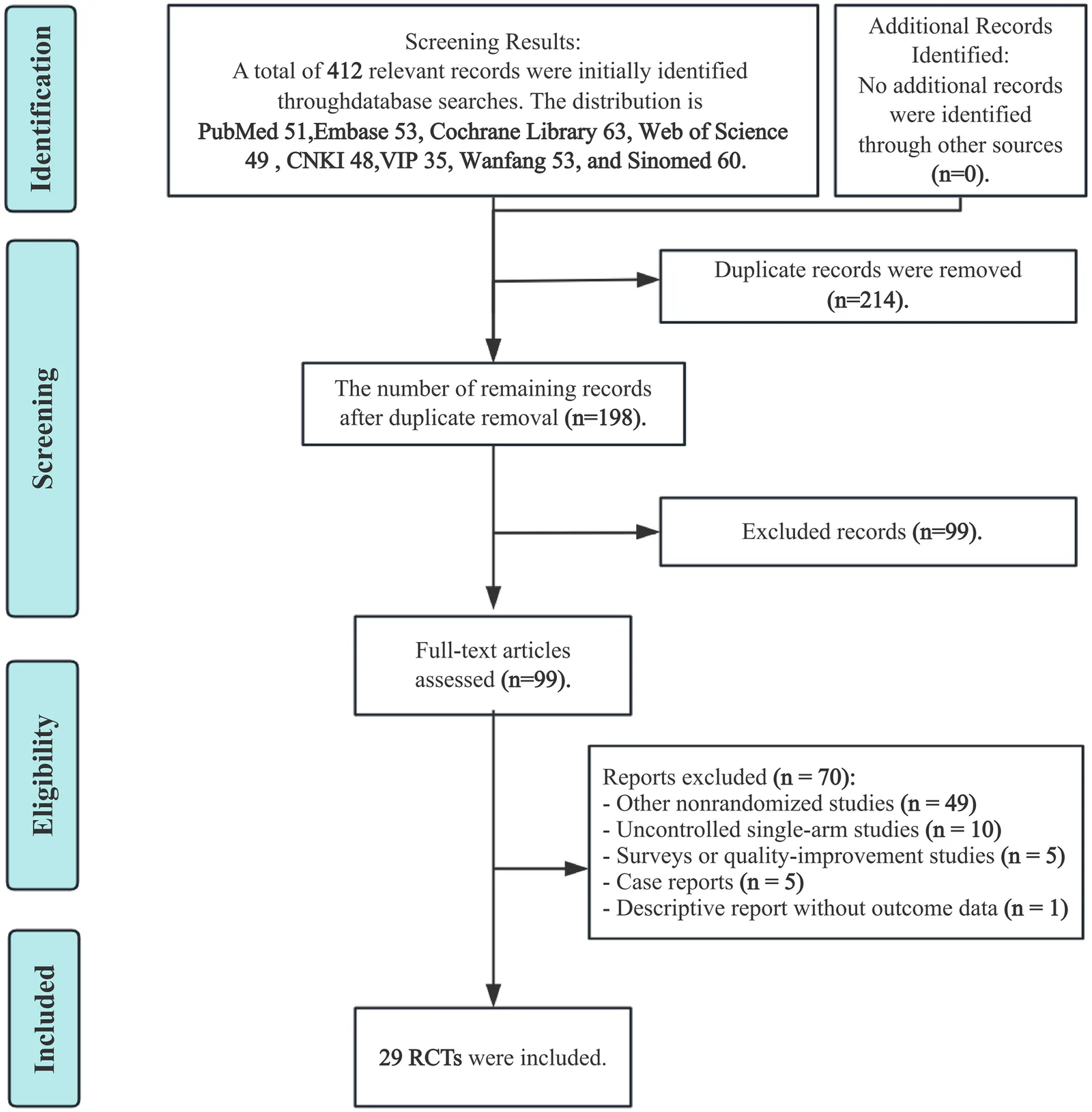
PRISMA 2020 flow diagram of study retrieval, screening, and inclusion.

### Characteristics of included studies

The 29 included studies were published between 1987 and 2026 and conducted across 11 countries, with 17 studies from China. The group sample sizes listed in Table 1 totalled 3,336 participants: 1,678 in the intervention groups and 1,658 in the control groups. Sample sizes ranged from 12 to 978 participants per comparison. Reported group mean ages ranged from 26.85 to 85.1 years. Twenty studies reported mean body-mass index, ranging from 22.06 to 30.90 kg/m^2^. Twenty studies involved surgical valve procedures, eight involved TAVR/TAVI, and one included both procedure types. Rehabilitation began preoperatively in four studies, perioperatively in three, and postoperatively in 22. Programmes included aerobic, resistance, respiratory, early mobilisation, and multimodal approaches, sometimes with education or psychological support. Structured courses ranged from brief inpatient training to several months. Comparators included usual care, standard rehabilitation, exercise advice, education, and sham training. Follow-up ranged from inpatient assessment to 12 months or 1 year after training. Table 1 summarises study characteristics. Detailed clinical baseline variables, including valve disease, sex, cardiac function, functional capacity, and frailty where reported, are provided in Supplementary Tables S3, S4. Intervention and comparator components, prescriptions, and timing are detailed in Supplementary Tables S5–S7, and selected functional assessments in Supplementary Table S8.

### Risk of bias assessment

Across the 29 trials, 83 selected study–outcome results were assessed using RoB 2: 17 were at high risk of bias and 66 raised some concerns; none was at overall low risk. Missing outcome data were the main source of high-risk judgments, with additional concerns about deviations from intended interventions. Randomisation and selection of the reported result frequently raised some concerns. Blinding participants and staff was often not feasible, but this did not by itself determine the overall judgment. Assessments concerned the effect of assignment to intervention for each selected outcome and assessment time. Outcome-specific judgments and supporting reasons are presented in Supplementary Table S11.

### Exercise capacity and cardiac function

Figure 2 summarises the effects of exercise-based rehabilitation on exercise capacity and cardiac function. Across 19 studies involving 1,574 participants, rehabilitation increased 6MWD by 40.95 m (95% CI 27.10 to 54.81; *P* < 0.001), with substantial heterogeneity (I^2^ = 90.1%; Figure 2A). Eight studies involving 518 participants assessed peak VO_2_. The pooled effect favoured rehabilitation (SMD 0.32, 95% CI 0.07 to 0.57; *P* = 0.014; I^2^ = 46.2%; Figure 2B). Fourteen studies involving 1,197 participants assessed LVEF, with a pooled increase of 4.41 percentage points (95% CI 2.26 to 6.56; *P* < 0.001; I^2^ = 89.5%; Figure 2C). The approximate 95% prediction intervals were −16.23 to 98.13 m for 6MWD, −0.33 to 0.97 for peak VO_2_, and −3.89 to 12.71 percentage points for LVEF; all included no effect (Supplementary Table S13).

**Figure 2:**
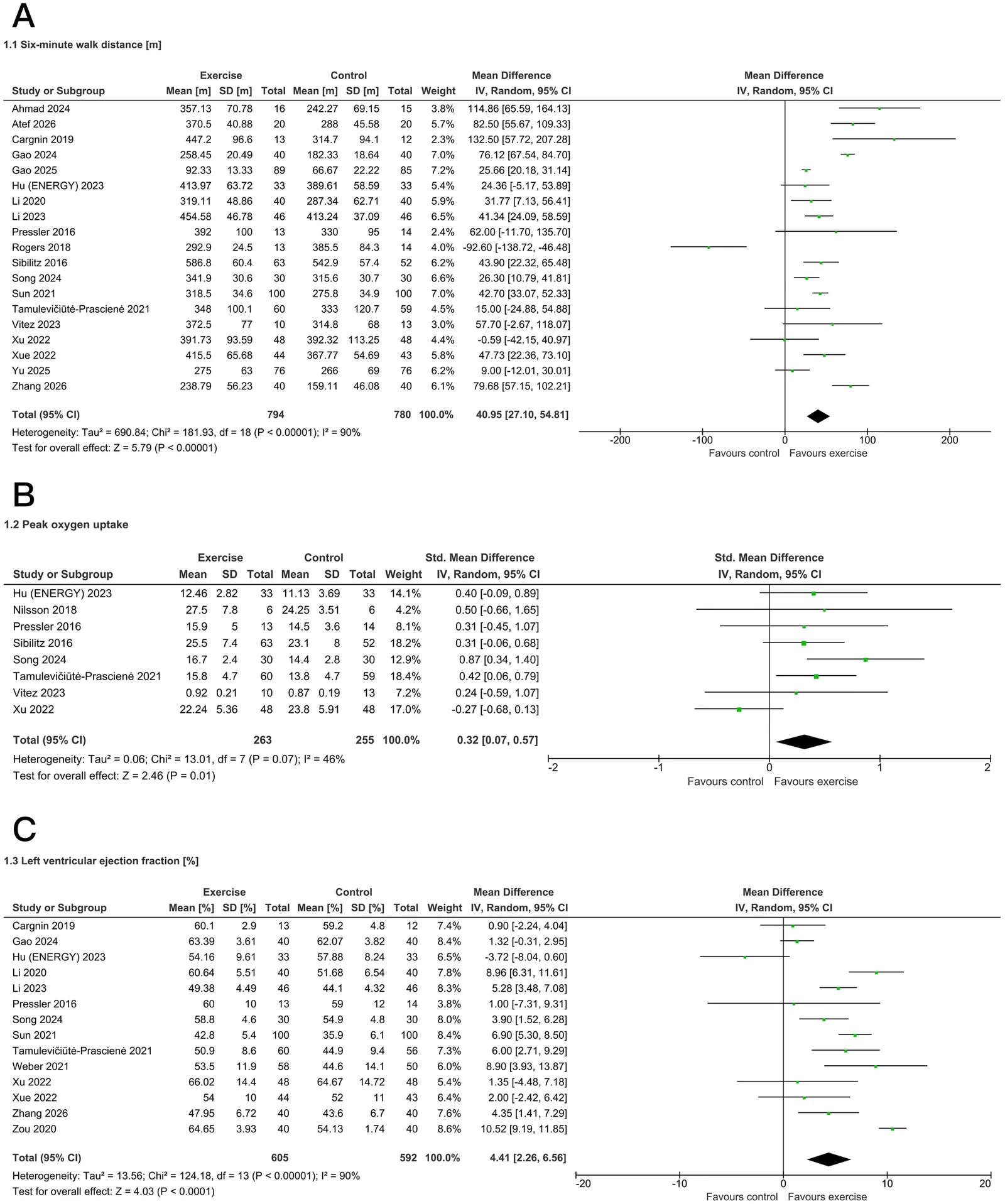
Effects of exercise-based rehabilitation on exercise capacity and cardiac function. Forest plots showing pooled random-effects estimates for **(A)** 6-min walk distance (m), **(B)** peak oxygen uptake, and **(C)** left ventricular ejection fraction (%). Continuous outcomes were pooled using inverse-variance random-effects models and are expressed as mean differences or standardised mean differences with 95% confidence intervals. CI, confidence interval; IV, inverse variance; SD, standard deviation; Std. Mean Difference, standardised mean difference.

### Postoperative recovery and reported adverse events

Figure 3 summarises postoperative recovery and reported events. Across 16 studies involving 2,327 participants, rehabilitation shortened postoperative hospital stay by 1.77 days (MD −1.77 days, 95% CI −2.41 to −1.13; *P* < 0.001; I^2^ = 90.8%; Figure 3A). Nineteen studies involving 1,438 participants reported adverse-event data. Five had no events in either group and did not contribute to the RR estimate; the remaining 14 studies involving 1,276 participants yielded an RR of 0.53 (95% CI 0.33 to 0.85; *P* = 0.009; I^2^ = 44.2%; Figure 3B). Seven studies involving 1,554 participants reported postoperative pulmonary complications, which were less frequent with rehabilitation (RR 0.62, 95% CI 0.50 to 0.77; *P* < 0.001; I^2^ = 17.9%; Figure 3C). The approximate 95% prediction intervals were −4.24 to 0.69 days for hospital stay, 0.14 to 1.99 for adverse events, and 0.42 to 0.93 for pulmonary complications (Supplementary Table S13). Adverse-event definitions and observation periods varied across studies, so the pooled estimate describes reported clinical events rather than a uniform measure of exercise-related harm (Supplementary Tables S9, S10).

**Figure 3:**
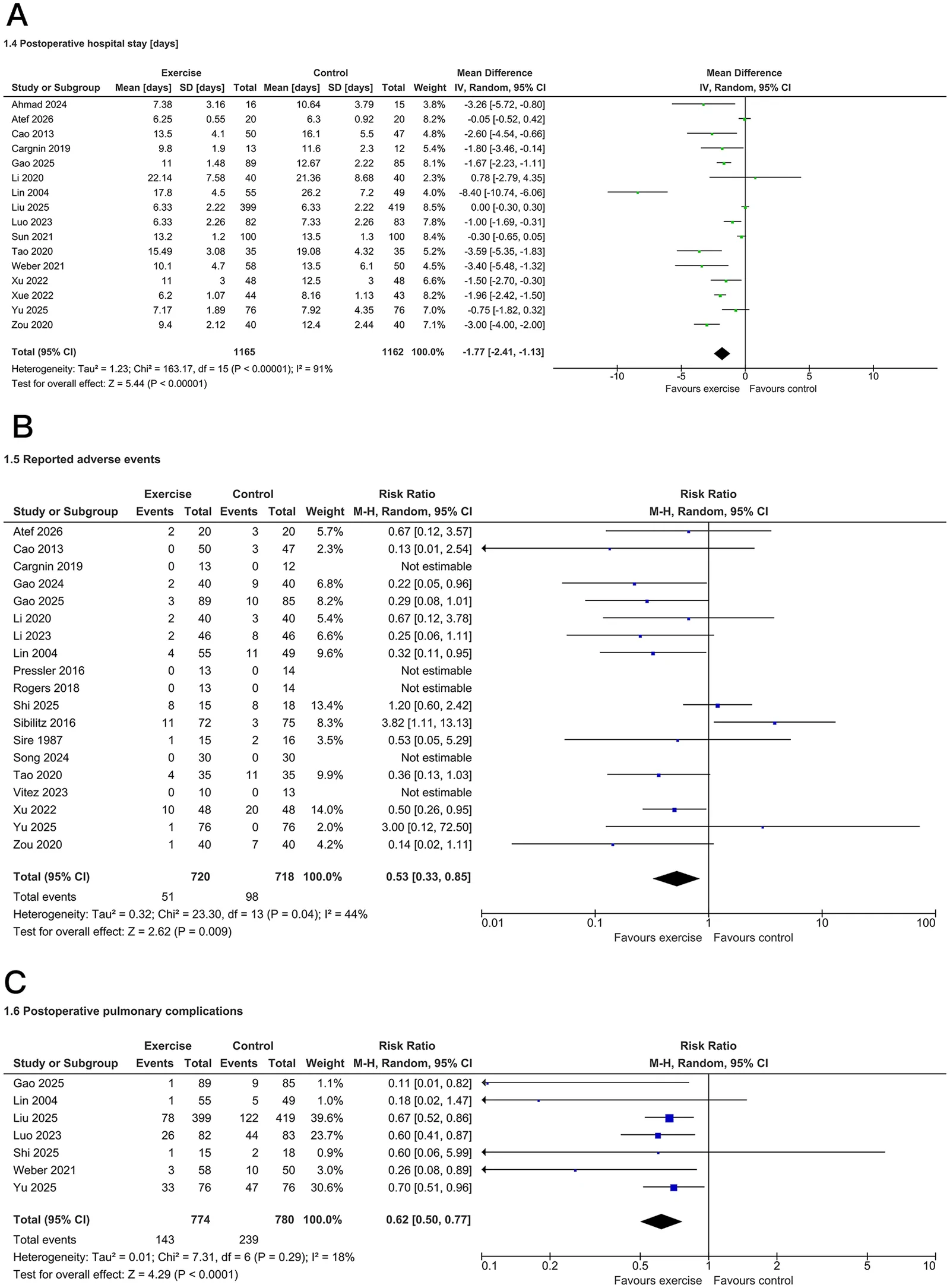
Effects of exercise-based rehabilitation on postoperative recovery and reported safety outcomes. Forest plots showing pooled random-effects estimates for **(A)** postoperative hospital stay (days), **(B)** reported adverse events, and **(C)** postoperative pulmonary complications. Postoperative hospital stay was analysed using inverse-variance mean differences, and binary outcomes were analysed using Mantel–Haenszel risk ratios with 95% confidence intervals. Studies with zero events in both groups were not estimable for risk ratios. CI, confidence interval; IV, inverse variance; M–H, Mantel–Haenszel; SD, standard deviation.

### Subgroup analysis

Subgroup analyses examined procedure type, structured intervention duration, and rehabilitation modality (Supplementary Table S14). For 6MWD, the estimated effect was larger after surgery (13 studies; MD 51.59 m, 95% CI 36.14 to 67.03) than after TAVR/TAVI (six studies; MD 10.20 m, −25.28 to 45.68), with a nominally significant test of subgroup differences (*P* = 0.036). Procedure-specific interaction tests were not statistically significant for peak VO_2_ (*P* = 0.768), LVEF (*P* = 0.246), hospital stay (*P* = 0.649), adverse events (*P* = 0.335), or pulmonary complications (*P* = 0.220).

Duration was associated with differences in hospital-stay estimates (*P* < 0.001), but the >8-week subgroup contained only one study. No significant duration interaction was found for the other outcomes. Modality interaction tests were significant for peak VO_2_ (*P* = 0.011), LVEF (*P* = 0.003), and hospital stay (*P* < 0.001), but not for 6MWD, adverse events, or pulmonary complications. Eighteen interaction tests were performed without adjustment for multiple comparisons. Several comparisons contained subgroups with fewer than three estimable studies, so these findings require cautious, exploratory interpretation.

### Sensitivity analyses, publication bias, and evidence certainty

Fixed-effect analyses retained the direction and statistical significance of all six principal results. Leave-one-out analyses retained the direction of every pooled effect; however, the peak VO_2_ result was no longer statistically significant after omitting Tamulevičiūtė-Prascienė 2021 (*P* = 0.055). Excluding high-risk results for each specific outcome retained the findings for 6MWD, hospital stay, adverse events, and pulmonary complications, whereas the peak VO_2_ estimate became imprecise and crossed the null (five studies; SMD 0.33, 95% CI −0.14 to 0.80; *P* = 0.170). No LVEF result was rated high risk, so no high-risk exclusion analysis was applicable to this outcome.

Timing-restricted analyses found an increase in 6MWD within 30 days of the procedure (three studies; MD 44.80 m, 95% CI 13.22 to 76.37), but the estimate beyond 90 days was imprecise (six studies; MD 13.97 m, −26.42 to 54.36). Beyond 90 days, peak VO_2_ favoured rehabilitation (seven studies; SMD 0.23, 0.01 to 0.46), whereas LVEF did not (three studies, all in TAVR populations; MD −1.34 percentage points, −4.84 to 2.16). No selected functional result could be assigned unambiguously to the 31–90-day window. These restrictions did not constitute formal comparisons between follow-up windows. An additional sensitivity analysis excluding the trial with uncertainty about the unit of randomisation retained the direction and statistical significance of all four affected outcomes. Detailed results are presented in Supplementary Table S15.

Funnel plots showed possible asymmetry for 6MWD and hospital stay and irregularity for LVEF, although heterogeneity could also explain these patterns. The adverse-event plot was inconclusive. Only eight studies assessed peak VO_2_ and seven assessed pulmonary complications, limiting interpretation of their funnel plots (Supplementary Figures S1–S6). Evidence certainty was very low for 6MWD, peak VO_2_, LVEF, hospital stay, and reported adverse events, and low for pulmonary complications. Outcome-specific downgrading reflected risk of bias, inconsistency, indirectness, imprecision, and suspected publication bias as applicable (Supplementary Table S16).

## Discussion

### Primary finding

This systematic review and meta-analysis included 29 randomised trials of exercise-based rehabilitation initiated before, around, or after surgical or transcatheter valve intervention. The principal analyses favoured rehabilitation for 6MWD, peak VO_2_, LVEF, postoperative hospital stay, reported adverse events, and pulmonary complications. However, substantial heterogeneity affected several outcomes, and prediction intervals crossed the null except for pulmonary complications. The peak VO_2_ finding was particularly sensitive to exclusion of high-risk results and one influential study. Procedure-specific interaction testing suggested a possible difference in 6MWD, but did not establish differences for the other outcomes. Evidence certainty was low for pulmonary complications and very low for all other outcomes. These findings indicate potential benefits across the valve intervention pathway, while leaving uncertainty about their magnitude, durability, and applicability to particular patients and programmes.

### Potential mechanisms

The benefits of exercise-based rehabilitation may reflect combined changes in the cardiovascular, respiratory, muscular, and nervous systems (40). Regular exercise improves blood-vessel function and circulation by increasing nitric oxide availability. It also supports mitochondrial function, energy production, and antioxidant activity through pathways such as AMPK/SIRT1/PGC-1*α* (40, 41). These changes help the body use oxygen more efficiently, reduce the workload on the heart, and improve exercise capacity. Aerobic exercise, alone or combined with strength training, may also improve ventricular filling, cardiac reserve, and heart-rate regulation, thereby supporting cardiac recovery after treatment (42, 43). Improved balance between sympathetic and parasympathetic activity might reduce excessive cardiac stress and susceptibility to abnormal heart rhythms (44). These effects could partly explain the observed improvements in left ventricular ejection fraction and physical function. Breathing exercises and early mobilisation may reduce pulmonary complications by strengthening the breathing muscles, improving ventilation, and supporting airway clearance (45). Progressive activity also limits the muscle loss and metabolic changes caused by prolonged bed rest, improving lower-limb strength and daily function (5). These mechanisms are plausible explanations, but were not directly tested by this meta-analysis.

### Interpretation of efficacy and safety outcomes

The pooled 40.95 m increase in 6MWD suggests a potential improvement in functional capacity, but its clinical meaning depends on baseline function, procedure, and assessment timing. The substantial heterogeneity and prediction interval spanning no benefit preclude assuming that a similar gain will occur in every setting. Walking distance is influenced by muscle strength, balance, symptoms, movement efficiency, and motivation, whereas peak VO_2_ more directly reflects the integrated cardiorespiratory response during exertion (17, 41, 44).

The principal peak VO_2_ analysis showed a small average benefit, but this was less secure than its *P* value alone suggests. Only eight studies involving 518 participants contributed, compared with 19 studies and 1,574 participants for 6MWD. Small trials and incomplete CPET follow-up reduce precision, as illustrated by the wide confidence interval after exclusion of high-risk results. Differences in workload progression, gas-exchange averaging, and criteria for maximal effort can also affect CPET estimates; included trials used individually selected cycle-ergometer increments or ramps based on estimated maximal workload (17, 30). Using an SMD accommodates differing reporting scales but does not remove these protocol differences. Older TAVR participants may stop because of fatigue, musculoskeletal limitations, or frailty before reaching maximal cardiorespiratory effort (30, 46). However, effort criteria and frailty were not reported consistently enough to test this explanation. Age alone does not establish that maximal testing was unsafe, and the present data cannot attribute the uncertain peak VO_2_ effect to any single mechanism.

Rehabilitation also increased LVEF in the principal analysis, but this finding should be interpreted cautiously. Ejection fraction varies with valve disease, preoperative cardiac function, procedure, measurement method, and follow-up duration (42). The high heterogeneity, prediction interval crossing no effect, and absence of a clear benefit in the >90-day analysis limit confidence in a consistent improvement. The latter analysis included only three TAVR studies and therefore cannot establish a decline in treatment effect over time. Patients can become more active and less symptomatic without a short-term change in resting ejection fraction, so this measure should be considered alongside exercise capacity, symptoms, quality of life, and clinical events (47, 48).

Rehabilitation shortened hospital stay and was associated with fewer pulmonary complications. Early activity, breathing exercises, and progressive training may support recovery, but discharge practices and accompanying care also influence these outcomes (3, 5, 38, 45). The pooled hospital-stay effect therefore should not be attributed solely to exercise. The reduction in reported adverse events requires still greater caution because studies combined exercise-associated symptoms, general postoperative complications, and broader clinical events over different observation periods. A lower pooled RR does not establish that rehabilitation itself is safer, nor do double-zero studies establish an absence of harm. Pulmonary complications had a more consistent pooled result, but variable diagnostic criteria and prevention packages, together with risk of bias, still limited certainty.

Earlier reviews were more cautious. A Cochrane review found uncertainty about important clinical outcomes after valve surgery because the evidence was of very low certainty (13). A TAVR-specific meta-analysis also reported no statistically significant improvements in 6MWD, peak VO_2_, or LVEF (8). The present review included additional randomised evidence and a broader range of perioperative rehabilitation strategies, yielding favourable average estimates across the six outcomes (3, 11, 12, 34–39). Differences in populations, comparators, and outcome timing can explain some disagreement between reviews. The larger evidence base does not remove these limitations or establish long-term effects on readmission or survival (4, 13).

### Interpretation of subgroup and sensitivity analyses

The formal interaction test suggested a larger 6MWD effect in surgical than in TAVR/TAVI studies. However, the nominal *P* value of 0.036 arose among 18 unadjusted comparisons and should be regarded as a signal for further study. TAVR populations are often older and have frailty or multiple coexisting conditions, while surgical populations may differ in baseline function and the burden of postoperative deconditioning (2, 46). These differences could affect rehabilitation response, but study-level comparisons cannot isolate procedure type from age, baseline function, programme content, or follow-up timing. For LVEF and the other outcomes, differences in within-subgroup significance did not translate into significant interaction tests and do not justify claims of greater benefit after surgery.

Both shorter and longer structured programmes had favourable 6MWD estimates, but the duration interaction was not significant. The hospital-stay interaction was driven by a comparison with only one >8-week study; moreover, hospital stay occurred before completion of the longer course, so this finding cannot establish a causal benefit of longer training. Modality interactions for peak VO_2_, LVEF, and hospital stay likewise involved sparse subgroups and overlapping programme components. Although aerobic, resistance, respiratory, and multimodal programmes have different aims, the present data do not identify an optimal modality or duration. Pooling provides an average effect of additional rehabilitation as delivered in the trials, and random-effects modelling does not make clinically different interventions and comparators interchangeable.

Sensitivity analyses supported the direction of the principal results, but highlighted uncertainty in peak VO_2_. The similar point estimate and wider interval after high-risk exclusions indicate reduced precision rather than proof that no benefit exists. For 6MWD and LVEF, later-window estimates were also imprecise, and restriction changed the contributing populations as well as assessment timing. These analyses cannot establish whether benefits diminish over time. Removing high-risk results or applying a fixed-effect model also cannot eliminate residual bias, indirectness, or the clinical heterogeneity underlying the very low certainty of most outcomes.

### Clinical implications

Exercise-based rehabilitation may support recovery across preoperative, perioperative, and postoperative care, with programmes tailored to clinical stability, procedure, baseline function, and patient goals (2, 6, 42). Inpatient care can include breathing exercises and progressive mobilisation, while post-discharge programmes may incorporate aerobic, resistance, and balance training (5, 46, 49). After open surgery, exercise prescription should account for wound healing, pain, and sternal stability. For older TAVR/TAVI patients, frailty, balance, leg strength, and independence in daily activities may be particularly relevant (2, 46). The present evidence does not establish a single optimal start time, training intensity, or duration. Clinical supervision and systematic recording of exercise-related symptoms and adverse events remain important, because the pooled clinical-event data are insufficient to establish exercise-specific safety.

### Strengths and limitations

This review included a broad range of randomised evidence and examined exercise capacity, cardiac function, postoperative recovery, and reported events. It assessed procedure-specific effects with formal interaction tests, used result-specific RoB 2 judgments, and examined analytical assumptions and outcome timing in sensitivity analyses. Several limitations remain. Studies differed in patient age, valve disease, procedure, rehabilitation timing and content, comparator care, and follow-up. The pooled effects therefore concern heterogeneous packages of additional rehabilitation and cannot isolate exercise from accompanying components. Baseline frailty, functional status, and adherence were incompletely reported, limiting exploration of effect modifiers. None of the 83 selected results was at overall low risk of bias.

Several analyses contained few studies, and the DerSimonian–Laird models with Wald intervals and approximate prediction intervals may provide imperfect uncertainty estimates in sparse or heterogeneous datasets. The 18 subgroup tests were not adjusted for multiplicity, and timing-restricted analyses were introduced during revision. Unclear assessment timing excluded many results from these restrictions, potentially changing the populations represented. Uncertainty about the allocation unit in one trial could not be resolved; its exclusion did not materially alter the affected findings, but cannot substitute for design clarification. Variable adverse-event definitions limited assessment of intervention-related harms. Possible publication bias, including asymmetry for hospital stay, could not be excluded. Historical search and screening records were incomplete, limiting verification of database-specific execution details and the relationship between the supplementary exclusion register and the reported study flow. Most studies provided limited long-term follow-up, leaving effects on readmission, major cardiovascular events, and survival uncertain.

### Future research

Future multicentre randomised trials should recruit sufficient participants, conceal treatment allocation, and use blinded outcome assessment where possible. Surgical and transcatheter populations should be studied separately, with greater attention to older patients and those with frailty or multiple conditions. Exercise programmes should clearly report their frequency, intensity, duration, type, total amount, and progression. Standard outcomes and assessment times are also needed for walking distance, peak oxygen uptake, ejection fraction, quality of life, frailty, and postoperative complications. Future studies should extend beyond short-term physical outcomes to assess readmission, major cardiovascular events, survival, long-term quality of life, health-care use, and cost-effectiveness. They should also report exercise-related adverse events, adherence, withdrawal reasons, and programme acceptability. Remote rehabilitation, wearable monitoring, and digital exercise programmes might improve access and long-term participation, especially for patients unable to attend centre-based rehabilitation. Their effectiveness and safety should be tested in well-designed trials.

## Conclusion

Exercise-based rehabilitation initiated before, around, or after surgical or transcatheter valve intervention may improve exercise capacity and cardiac function, shorten hospital stay, and reduce reported clinical events and pulmonary complications. However, evidence certainty was very low for five outcomes and low for pulmonary complications. The peak VO_2_ finding was sensitive to high-risk exclusions, and prediction intervals for most outcomes included no benefit. The evidence does not establish exercise-specific safety or an optimal rehabilitation programme. Larger randomised trials with clearly described interventions and comparators, consistent assessment times, and longer follow-up are needed.
