# Supplementary material for: Efficacy and safety of exercise rehabilitation across the valve intervention pathway: A systematic review and meta-analysis
Source: Front Cardiovasc Med. 2026 Oct 29;13:2004830. doi: 10.3389/fcvm.2026.2004830 (PMC13755833; doi:10.3389/fcvm.2026.2004830)
Supplement: Supplementary file 1 [file Table1.docx]

Supplementary Appendix

# Contents

Appendix 1	PRISMA 2020 checklist	S2

Appendix 2	Database search strategies	S5

Appendix 3	Study selection and full text exclusions	S7

Appendix 4	Study populations and baseline characteristics	S15

Appendix 5	Intervention and comparator details	S19

Appendix 6	Rehabilitation stages and outcome assessment timing	S25

Appendix 7	Safety outcome definitions	S30

Appendix 8	Outcome specific risk of bias	S33

Appendix 9	Study grouping for subgroup analyses	S37

Appendix 10	Main analysis estimates and prediction intervals	S39

Appendix 11	Subgroup and sensitivity analyses	S40

Appendix 12	Funnel plots	S43

Appendix 13	GRADE evidence profile	S46

# Appendix 1 PRISMA 2020 checklist

## Table S1 PRISMA 2020 checklist

| **Section and topic** | **Item** | **Checklist item** | **Location or outstanding item** |
| --- | --- | --- | --- |
| Title | 1 | Identify the report as a systematic review. | Manuscript title. |
| Abstract | 2 | See the PRISMA 2020 for Abstracts checklist. | Manuscript structured abstract. |
| Rationale | 3 | Describe the rationale for the review in the context of existing knowledge. | Manuscript Introduction. |
| Objectives | 4 | Provide an explicit statement of the objective(s) or question(s) the review addresses. | Introduction; scope includes preoperative, perioperative and postoperative rehabilitation. |
| Eligibility criteria | 5 | Specify the inclusion and exclusion criteria for the review and how studies were grouped for the syntheses. | Methods, Inclusion and exclusion criteria; Tables S5–S8 and S12. |
| Information sources | 6 | Specify all databases, registers, websites, organisations, reference lists and other sources searched or consulted to identify studies. Specify the date when each source was last searched or consulted. | Appendix 2 |
| Search strategy | 7 | Present the full search strategies for all databases, registers and websites, including any filters and limits used. | Appendix 2 |
| Selection process | 8 | Specify the methods used to decide whether a study met the inclusion criteria of the review, including how many reviewers screened each record and each report retrieved, whether they worked independently, and if applicable, details of automation tools used in the process. | Methods, Study selection and data extraction |
| Data collection process | 9 | Specify the methods used to collect data from reports, including how many reviewers collected data from each report, whether they worked independently, any processes for obtaining or confirming data from study investigators, and if applicable, details of automation tools used in the process. | Methods, Study selection and data extraction; independent review and disagreement resolution are described by the authors. |
| Data items | 10a | List and define all outcomes for which data were sought. Specify whether all results that were compatible with each outcome domain in each study were sought (e.g. for all measures, time points, analyses), and if not, the methods used to decide which results to collect. | Appendices 6 and 7; selected results and explicit timing windows. |
| Data items | 10b | List and define all other variables for which data were sought (e.g. participant and intervention characteristics, funding sources). Describe any assumptions made about any missing or unclear information. | Appendices 4 and 5. |
| Study risk of bias assessment | 11 | Specify the methods used to assess risk of bias in the included studies, including details of the tool(s) used, how many reviewers assessed each study and whether they worked independently, and if applicable, details of automation tools used in the process. | Methods, Risk-of-bias assessment; Appendix 8, Table S11; assignment estimand and result-specific judgments. |
| Effect measures | 12 | Specify for each outcome the effect measure(s) (e.g. risk ratio, mean difference) used in the synthesis or presentation of results. | Appendix 10; MD, SMD and RR. |
| Synthesis methods | 13a | Describe the processes used to decide which studies were eligible for each synthesis (e.g. tabulating the study intervention characteristics and comparing against the planned groups for each synthesis (item #5)). | Appendices 5, 6 and 9. |
| Synthesis methods | 13b | Describe any methods required to prepare the data for presentation or synthesis, such as handling of missing summary statistics, or data conversions. | Methods, Study selection and data extraction; conversions are described. Study-specific conversion records are not tabulated. |
| Synthesis methods | 13c | Describe any methods used to tabulate or visually display results of individual studies and syntheses. | Manuscript Table 1 and Figures 1–3; Tables S11 and S13–S15; Figures S1–S6. |
| Synthesis methods | 13d | Describe any methods used to synthesize results and provide a rationale for the choice(s). If meta-analysis was performed, describe the model(s), method(s) to identify the presence and extent of statistical heterogeneity, and software package(s) used. | Appendices 10 and 11. |
| Synthesis methods | 13e | Describe any methods used to explore possible causes of heterogeneity among study results (e.g. subgroup analysis, meta-regression). | Appendices 9 and 11. |
| Synthesis methods | 13f | Describe any sensitivity analyses conducted to assess robustness of the synthesized results. | Appendix 11. |
| Reporting bias assessment | 14 | Describe any methods used to assess risk of bias due to missing results in a synthesis (arising from reporting biases). | Appendix 12; visual funnel assessment. |
| Certainty assessment | 15 | Describe any methods used to assess certainty (or confidence) in the body of evidence for an outcome. | Methods, Certainty of evidence assessment; Table S16 and outcome-specific explanations. |
| Study selection | 16a | Describe the results of the search and selection process, from the number of records identified in the search to the number of studies included in the review, ideally using a flow diagram. | Results, Literature screening and included studies; Figure 1. |
| Study selection | 16b | Cite studies that might appear to meet the inclusion criteria, but which were excluded, and explain why they were excluded. | Appendix 3, Table S2: 70 supplementary full-text exclusions; |
| Study characteristics | 17 | Cite each included study and present its characteristics. | Manuscript Table 1 with citations for all 29 trials; Tables S3–S8. |
| Risk of bias in studies | 18 | Present assessments of risk of bias for each included study. | Appendix 8. |
| Results of individual studies | 19 | For all outcomes, present, for each study: (a) summary statistics for each group (where appropriate) and (b) an effect estimate and its precision (e.g. confidence/credible interval), ideally using structured tables or plots. | Figures 2A–2C and 3A–3C show group data and individual effect estimates. |
| Results of syntheses | 20a | For each synthesis, briefly summarise the characteristics and risk of bias among contributing studies. | Appendices 4–9. |
| Results of syntheses | 20b | Present results of all statistical syntheses conducted. If meta-analysis was done, present for each the summary estimate and its precision (e.g. confidence/credible interval) and measures of statistical heterogeneity. If comparing groups, describe the direction of the effect. | Appendices 10 and 11. |
| Results of syntheses | 20c | Present results of all investigations of possible causes of heterogeneity among study results. | Appendix 11, Table S14. |
| Results of syntheses | 20d | Present results of all sensitivity analyses conducted to assess the robustness of the synthesized results. | Appendix 11, Table S15. |
| Reporting biases | 21 | Present assessments of risk of bias due to missing results (arising from reporting biases) for each synthesis assessed. | Appendix 12; synthesis-level reporting-bias judgments are described in Appendix 13. |
| Certainty of evidence | 22 | Present assessments of certainty (or confidence) in the body of evidence for each outcome assessed. | Appendix 13, Table S16 and outcome-specific certainty explanations. |
| Discussion | 23a | Provide a general interpretation of the results in the context of other evidence. | Revised manuscript Discussion. |
| Discussion | 23b | Discuss any limitations of the evidence included in the review. | Revised manuscript Discussion and Strengths and limitations. |
| Discussion | 23c | Discuss any limitations of the review processes used. | Discussion, Strengths and limitations; incomplete historical search and screening records are acknowledged. |
| Discussion | 23d | Discuss implications of the results for practice, policy, and future research. | Revised manuscript Clinical implications and Future research. |
| Registration and protocol | 24a | Provide registration information for the review, including register name and registration number, or state that the review was not registered. | PROSPERO CRD420261509016. |
| Registration and protocol | 24b | Indicate where the review protocol can be accessed, or state that a protocol was not prepared. | PROSPERO CRD420261509016. |
| Registration and protocol | 24c | Describe and explain any amendments to information provided at registration or in the protocol. | PROSPERO CRD420261509016 |
| Support | 25 | Describe sources of financial or non-financial support for the review, and the role of the funders or sponsors in the review. | Manuscript funding statement |
| Competing interests | 26 | Declare any competing interests of review authors. | Manuscript conflict of interest statement. |
| Availability of data, code and other materials | 27 | Report which of the following are publicly available and where they can be found: template data collection forms; data extracted from included studies; data used for all analyses; analytic code; any other materials used in the review. | Manuscript Data availability statement describes access on request. No public data or code repository is specified. |

# Appendix 2 Database search strategies

## PubMed

#1("Heart Valve Diseases"[Mesh] OR "Valvular Heart Disease"[Title/Abstract] OR "Valvular Heart Diseases"[Title/Abstract] OR "Heart Valve Disease"[Title/Abstract] OR "Aortic Valve Stenosis"[Title/Abstract] OR "Mitral Valve Disease"[Title/Abstract])

#2("Heart Valve Prosthesis Implantation"[Mesh] OR "Heart Valve Surgery"[Title/Abstract] OR "Valve Surgery"[Title/Abstract] OR "Valve Replacement"[Title/Abstract] OR "Valve Repair"[Title/Abstract] OR "Surgical Aortic Valve Replacement"[Title/Abstract] OR SAVR[Title/Abstract] OR "Transcatheter Aortic Valve Replacement"[Title/Abstract] OR "Transcatheter Aortic Valve Implantation"[Title/Abstract] OR TAVR[Title/Abstract] OR TAVI[Title/Abstract])

#3("Cardiac Rehabilitation"[Mesh] OR "Exercise Therapy"[Mesh] OR "Exercise"[Mesh] OR "Cardiac Rehabilitation"[Title/Abstract] OR "Exercise Rehabilitation"[Title/Abstract] OR "Exercise Training"[Title/Abstract] OR "Aerobic Exercise"[Title/Abstract] OR "Resistance Training"[Title/Abstract] OR "Inspiratory Muscle Training"[Title/Abstract] OR "Respiratory Training"[Title/Abstract] OR "Early Mobilization"[Title/Abstract] OR Prehabilitation[Title/Abstract])

#4("Randomized Controlled Trial"[Publication Type] OR "Controlled Clinical Trial"[Publication Type] OR random*[Title/Abstract] OR trial[Title/Abstract])

#5 #1 AND #2 AND #3 AND #4

## Embase.com (Elsevier)

#1 'heart valve disease'/exp OR ('valvular heart disease' OR 'valvular heart diseases' OR 'heart valve disease' OR 'heart valve diseases' OR 'aortic valve stenosis' OR 'mitral valve disease'):ti,ab,kw

#2 'heart valve replacement'/exp OR 'heart valve surgery'/exp OR ('heart valve surgery' OR 'valve surgery' OR 'valve replacement' OR 'valve repair' OR 'surgical aortic valve replacement' OR SAVR OR 'transcatheter aortic valve replacement' OR 'transcatheter aortic valve implantation' OR TAVR OR TAVI):ti,ab,kw

#3 'heart rehabilitation'/exp OR 'kinesiotherapy'/exp OR 'exercise'/exp OR ('cardiac rehabilitation' OR 'exercise rehabilitation' OR 'exercise therapy' OR exercis* OR 'exercise training' OR 'aerobic exercise' OR 'resistance training' OR 'inspiratory muscle training' OR 'respiratory training' OR 'early mobilization' OR 'early mobilisation' OR prehabilitation):ti,ab,kw

#4 'randomized controlled trial'/exp OR 'controlled clinical trial'/exp OR (random* OR trial*):ti,ab,kw

#5 #1 AND #2 AND #3 AND #4

## Web of Science Core Collection

#1 TS=("valvular heart disease" OR "valvular heart diseases" OR "heart valve disease" OR "heart valve diseases" OR "aortic valve stenosis" OR "mitral valve disease")

#2 TS=("heart valve surgery" OR "valve surgery" OR "valve replacement" OR "valve repair" OR "surgical aortic valve replacement" OR SAVR OR "transcatheter aortic valve replacement" OR "transcatheter aortic valve implantation" OR TAVR OR TAVI)

#3 TS=("cardiac rehabilitation" OR "exercise rehabilitation" OR "exercise therapy" OR exercis* OR "exercise training" OR "aerobic exercise" OR "resistance training" OR "inspiratory muscle training" OR "respiratory training" OR "early mobilization" OR "early mobilisation" OR prehabilitation)

#4 TS=(random* OR trial* OR "randomized controlled trial" OR "randomised controlled trial" OR "controlled clinical trial")

#5 #1 AND #2 AND #3 AND #4

## Cochrane Central Register of Controlled Trials

#1 MeSH descriptor: [Heart Valve Diseases] explode all trees

#2 ("valvular heart disease" OR "valvular heart diseases" OR "heart valve disease" OR "heart valve diseases" OR "aortic valve stenosis" OR "mitral valve disease"):ti,ab,kw

#3 #1 OR #2

#4 MeSH descriptor: [Heart Valve Prosthesis Implantation] explode all trees

#5 ("heart valve surgery" OR "valve surgery" OR "valve replacement" OR "valve repair" OR "surgical aortic valve replacement" OR SAVR OR "transcatheter aortic valve replacement" OR "transcatheter aortic valve implantation" OR TAVR OR TAVI):ti,ab,kw

#6 #4 OR #5

#7 MeSH descriptor: [Cardiac Rehabilitation] explode all trees

#8 MeSH descriptor: [Exercise Therapy] explode all trees

#9 MeSH descriptor: [Exercise] explode all trees

#10 ("cardiac rehabilitation" OR "exercise rehabilitation" OR "exercise therapy" OR exercis* OR "exercise training" OR "aerobic exercise" OR "resistance training" OR "inspiratory muscle training" OR "respiratory training" OR "early mobilization" OR "early mobilisation" OR prehabilitation):ti,ab,kw

#11 #7 OR #8 OR #9 OR #10

#12 #3 AND #6 AND #11

## CNKI, WanFang Data, VIP and SinoMed Shared Chinese search concepts

C1 (瓣膜性心脏病 OR 心脏瓣膜病 OR 心脏瓣膜疾病 OR 心瓣膜病 OR 主动脉瓣狭窄 OR 二尖瓣病)

C2 (心脏瓣膜手术 OR 瓣膜手术 OR 瓣膜置换 OR 瓣膜修复 OR 瓣膜成形 OR 外科主动脉瓣置换 OR 经导管主动脉瓣置换 OR 经导管主动脉瓣植入 OR SAVR OR TAVR OR TAVI)

C3 (心脏康复 OR 运动康复 OR 运动疗法 OR 运动训练 OR 有氧运动 OR 抗阻训练 OR 吸气肌训练 OR 呼吸肌训练 OR 呼吸训练 OR 呼吸锻炼 OR 早期活动 OR 早期下床 OR 预康复)

C4 (随机 OR 随机对照 OR 临床试验 OR 对照试验 OR RCT)

C5 C1 AND C2 AND C3 AND C4

# Appendix 3 Study selection and full text exclusions

## Table S2 Full text reports excluded after eligibility assessment

| **No.** | **Report and source** | **Specific primary reason for exclusion** |
| --- | --- | --- |
| 1 | **Meurin P, Iliou MC, Ben Driss A, et al.** Early exercise training after mitral valve repair: a multicentric prospective French study. Chest. 2005;128(3):1638-1644.  [DOI: 10.1378/chest.128.3.1638](https://doi.org/10.1378/chest.128.3.1638) \| [PMID 16162769](https://pubmed.ncbi.nlm.nih.gov/16162769/) | Nonrandomized single-arm cohort; patients were assessed before and after early exercise training following mitral valve repair. |
| 2 | **Nakamura K, Nakamura E, Niina K, et al.** Outcome after valve surgery in octogenarians and efficacy of early mobilization with early cardiac rehabilitation. Gen Thorac Cardiovasc Surg. 2010;58(12):606-611.  [DOI: 10.1007/s11748-010-0665-0](https://doi.org/10.1007/s11748-010-0665-0) \| [PMID 21170626](https://pubmed.ncbi.nlm.nih.gov/21170626/) | Retrospective cohort comparing age groups and postoperative recovery; rehabilitation was not randomly allocated. |
| 3 | **Pressler A, Scherr J, Eichinger W, et al.** Left ventricular remodeling with intensive exercise after aortic valve replacement. J Heart Valve Dis. 2011;20(1):91-93.  [PMID 21404903](https://pubmed.ncbi.nlm.nih.gov/21404903/) | Single-patient case report of intensive endurance exercise after aortic valve replacement. |
| 4 | **Strens D, Colle A, Vrijens F, et al.** Multidisciplinary outpatient rehabilitation following cardiac revascularization or valve surgery: patient-related factors for uptake. Eur J Prev Cardiol. 2013;20(3):422-430.  [DOI: 10.1177/2047487312441727](https://doi.org/10.1177/2047487312441727) \| [PMID 22392164](https://pubmed.ncbi.nlm.nih.gov/22392164/) | Observational claims analysis of rehabilitation uptake after cardiac procedures; no randomized rehabilitation comparison. |
| 5 | **Pardaens S, Moerman V, Willems AM, et al.** Impact of the preoperative risk and the type of surgery on exercise capacity and training after valvular surgery. Am J Cardiol. 2014;113(8):1383-1389.  [DOI: 10.1016/j.amjcard.2014.01.413](https://doi.org/10.1016/j.amjcard.2014.01.413) \| [PMID 24576546](https://pubmed.ncbi.nlm.nih.gov/24576546/) | Prospective cohort stratified by operative risk and surgical approach; no random allocation of rehabilitation. |
| 6 | **Russo N, Compostella L, Tarantini G, et al.** Cardiac rehabilitation after transcatheter versus surgical prosthetic valve implantation for aortic stenosis in the elderly. Eur J Prev Cardiol. 2014;21(11):1341-1348.  [DOI: 10.1177/2047487313494029](https://doi.org/10.1177/2047487313494029) \| [PMID 23757283](https://pubmed.ncbi.nlm.nih.gov/23757283/) | Nonrandomized comparison of TAVI and surgical valve recipients; both groups received rehabilitation. |
| 7 | **Zanettini R, Gatto G, Mori I, et al.** Cardiac rehabilitation and mid-term follow-up after transcatheter aortic valve implantation. J Geriatr Cardiol. 2014;11(4):279-285.  [DOI: 10.11909/j.issn.1671-5411.2014.04.001](https://doi.org/10.11909/j.issn.1671-5411.2014.04.001) \| [PMID 25593575](https://pubmed.ncbi.nlm.nih.gov/25593575/) | Prospective single-arm TAVI cohort with pre/post rehabilitation assessments; no randomized control group. |
| 8 | **Franco SS, Malbouisson LM, Grinberg M, et al.** A propose of pulmonary dysfunction stratification after valve surgery by physiotherapeutic assistance level. Rev Bras Cir Cardiovasc. 2015;30(2):188-197.  [DOI: 10.5935/1678-9741.20150006](https://doi.org/10.5935/1678-9741.20150006) \| [PMID 26107450](https://pubmed.ncbi.nlm.nih.gov/26107450/) | Nonrandomized physiotherapy study; treatment intensity was assigned by pulmonary impairment, not by random allocation. |
| 9 | **Goel K, Pack QR, Lahr B, et al.** Cardiac rehabilitation is associated with reduced long-term mortality in patients undergoing combined heart valve and CABG surgery. Eur J Prev Cardiol. 2015;22(2):159-168.  [DOI: 10.1177/2047487313512219](https://doi.org/10.1177/2047487313512219) \| [PMID 24265289](https://pubmed.ncbi.nlm.nih.gov/24265289/) | Observational cohort using propensity adjustment to compare rehabilitation participation after combined valve and CABG surgery. |
| 10 | **Hansen TB, Berg SK, Sibilitz KL, et al.** Availability of, referral to and participation in exercise-based cardiac rehabilitation after heart valve surgery: Results from the national CopenHeart survey. Eur J Prev Cardiol. 2015;22(6):710-718.  [DOI: 10.1177/2047487314536364](https://doi.org/10.1177/2047487314536364) \| [PMID 24857890](https://pubmed.ncbi.nlm.nih.gov/24857890/) | Nationwide survey of rehabilitation availability, referral and participation; not a randomized trial. |
| 11 | **Hansen TB, Zwisler AD, Berg SK, et al.** Exercise-based cardiac rehabilitation after heart valve surgery: cost analysis of healthcare use and sick leave. Open Heart. 2015;2(1):e000288.  [DOI: 10.1136/openhrt-2015-000288](https://doi.org/10.1136/openhrt-2015-000288) \| [PMID 26301099](https://pubmed.ncbi.nlm.nih.gov/26301099/) | Observational cost analysis using survey-reported rehabilitation participation; not the CopenHeartVR randomized trial. |
| 12 | **Savage PD, Rengo JL, Menzies KE, et al.** Cardiac Rehabilitation After Heart Valve Surgery: Comparison with coronary artery bypass graft patients. J Cardiopulm Rehabil Prev. 2015;35(4):231-237.  [DOI: 10.1097/hcr.0000000000000104](https://doi.org/10.1097/hcr.0000000000000104) \| [PMID 25622220](https://pubmed.ncbi.nlm.nih.gov/25622220/) | Prospective cohort of rehabilitation participants compared by surgical category; rehabilitation was not randomized. |
| 13 | **Völler H, Salzwedel A, Nitardy A, et al.** Effect of cardiac rehabilitation on functional and emotional status in patients after transcatheter aortic-valve implantation. Eur J Prev Cardiol. 2015;22(5):568-574.  [DOI: 10.1177/2047487314526072](https://doi.org/10.1177/2047487314526072) \| [PMID 24577878](https://pubmed.ncbi.nlm.nih.gov/24577878/) | Retrospective cohort comparing TAVI and surgical valve recipients referred to rehabilitation; no randomized rehabilitation allocation. |
| 14 | **Pack QR, Lahr BD, Squires RW, et al.** Survey Reported Participation in Cardiac Rehabilitation and Survival After Mitral or Aortic Valve Surgery. Am J Cardiol. 2016;117(12):1985-1991.  [DOI: 10.1016/j.amjcard.2016.03.052](https://doi.org/10.1016/j.amjcard.2016.03.052) \| [PMID 27138188](https://pubmed.ncbi.nlm.nih.gov/27138188/) | Observational cohort using survey-reported rehabilitation attendance and propensity adjustment; no random allocation. |
| 15 | **Beatty AL, Bradley SM, Maynard C, et al.** Referral to Cardiac Rehabilitation After Percutaneous Coronary Intervention, Coronary Artery Bypass Surgery, and Valve Surgery: Data From the Clinical Outcomes Assessment Program. Circ Cardiovasc Qual Outcomes. 2017;10(6):e003364.  [DOI: 10.1161/circoutcomes.116.003364](https://doi.org/10.1161/circoutcomes.116.003364) \| [PMID 28619725](https://pubmed.ncbi.nlm.nih.gov/28619725/) | Observational registry analysis of rehabilitation referral across hospitals; no randomized exercise comparison. |
| 16 | **Eichler S, Salzwedel A, Reibis R, et al.** Multicomponent cardiac rehabilitation in patients after transcatheter aortic valve implantation: Predictors of functional and psychocognitive recovery. Eur J Prev Cardiol. 2017;24(3):257-264.  [DOI: 10.1177/2047487316679527](https://doi.org/10.1177/2047487316679527) \| [PMID 27852810](https://pubmed.ncbi.nlm.nih.gov/27852810/) | Prospective rehabilitation cohort examining predictors of recovery after TAVI; no randomized control group. |
| 17 | **Pollmann AGE, Frederiksen M, Prescott E.** Cardiac Rehabilitation After Heart Valve Surgery: Improvement in exercise capacity and morbidity. J Cardiopulm Rehabil Prev. 2017;37(3):191-198.  [DOI: 10.1097/hcr.0000000000000208](https://doi.org/10.1097/hcr.0000000000000208) \| [PMID 27755256](https://pubmed.ncbi.nlm.nih.gov/27755256/) | Retrospective medical-record review comparing rehabilitation attenders and nonattenders; not a randomized trial. |
| 18 | **Solomon TR, DeJong S, Bilbrey T, et al.** Cardiac rehabilitation for a skydiver after aortic valve replacement for pure aortic regurgitation and resection of the ascending aorta complicated by active infective endocarditis and heart block requiring a pacemaker. Proc (Bayl Univ Med Cent). 2017;30(2):234-236.  [DOI: 10.1080/08998280.2017.11929601](https://doi.org/10.1080/08998280.2017.11929601) \| [PMID 28405096](https://pubmed.ncbi.nlm.nih.gov/28405096/) | Single-patient case report of tailored rehabilitation for return to skydiving after aortic valve surgery. |
| 19 | **Tarro Genta F, Tidu M, Bouslenko Z, et al.** Cardiac rehabilitation after transcatheter aortic valve implantation compared to patients after valve replacement. J Cardiovasc Med (Hagerstown). 2017;18(2):114-120.  [DOI: 10.2459/jcm.0000000000000494](https://doi.org/10.2459/jcm.0000000000000494) \| [PMID 27941588](https://pubmed.ncbi.nlm.nih.gov/27941588/) | Nonrandomized comparison of TAVI and surgical valve recipients, all receiving residential rehabilitation. |
| 20 | **Waite I, Deshpande R, Baghai M, et al.** Home-based preoperative rehabilitation (prehab) to improve physical function and reduce hospital length of stay for frail patients undergoing coronary artery bypass graft and valve surgery. J Cardiothorac Surg. 2017;12(1):91.  [DOI: 10.1186/s13019-017-0655-8](https://doi.org/10.1186/s13019-017-0655-8) \| [PMID 29073924](https://pubmed.ncbi.nlm.nih.gov/29073924/) | Nonrandomized single-arm prehabilitation pilot; participants enrolled voluntarily and had pre/post assessments. |
| 21 | **Butter C, Groß J, Haase-Fielitz A, et al.** Impact of Rehabilitation on Outcomes after TAVI: A Preliminary Study. J Clin Med. 2018;7(10):E326.  [DOI: 10.3390/jcm7100326](https://doi.org/10.3390/jcm7100326) \| [PMID 30301135](https://pubmed.ncbi.nlm.nih.gov/30301135/) | Observational cohort comparing rehabilitation recipients with patients who declined rehabilitation; no random allocation. |
| 22 | **Imran HM, Baig M, Mujib M, et al.** Comparison of phase 2 cardiac rehabilitation outcomes between patients after transcatheter versus surgical aortic valve replacement. Eur J Prev Cardiol. 2018;25(15):1577-1584.  [DOI: 10.1177/2047487318792099](https://doi.org/10.1177/2047487318792099) \| [PMID 30086685](https://pubmed.ncbi.nlm.nih.gov/30086685/) | Retrospective cohort comparing rehabilitation uptake and outcomes after TAVR versus surgical valve replacement. |
| 23 | **Jenkins N, Adams J, Bilbrey T, et al.** Specificity of training in cardiac rehabilitation to facilitate a patient's return to strenuous work following aortic valve replacement. Proc (Bayl Univ Med Cent). 2018;31(1):72-75.  [DOI: 10.1080/08998280.2017.1401843](https://doi.org/10.1080/08998280.2017.1401843) \| [PMID 29686560](https://pubmed.ncbi.nlm.nih.gov/29686560/) | Single-patient case report of work-specific rehabilitation after aortic valve replacement. |
| 24 | **Modica M, Castiglioni P, Minotti A, et al.** Psychological Profile in Coronary Artery By-Pass Graft Patients vs. Valve Replacement Patients Entering Cardiac Rehabilitation after Surgery. Sci Rep. 2018;8(1):14381.  [DOI: 10.1038/s41598-018-32696-5](https://doi.org/10.1038/s41598-018-32696-5) \| [PMID 30258180](https://pubmed.ncbi.nlm.nih.gov/30258180/) | Observational comparison of psychological profiles at rehabilitation entry after CABG or valve replacement; no randomized intervention. |
| 25 | **Vitale G, Sarullo S, Vassallo L, et al.** Prognostic Value of the 6-Min Walk Test After Open-Heart Valve Surgery: Experience of a cardiovascular rehabilitation program. J Cardiopulm Rehabil Prev. 2018;38(5):304-308.  [DOI: 10.1097/hcr.0000000000000340](https://doi.org/10.1097/hcr.0000000000000340) \| [PMID 29952806](https://pubmed.ncbi.nlm.nih.gov/29952806/) | Retrospective prognostic cohort assessing the six-minute walk test among rehabilitation participants; not a randomized trial. |
| 26 | **Kato M, Saitoh M, Kawamura T, et al.** Postoperative atrial fibrillation is associated with delayed early rehabilitation after heart valve surgery: a multicenter study. Phys Ther Res. 2019;22(1):1-8.  [DOI: 10.1298/ptr.e9957](https://doi.org/10.1298/ptr.e9957) \| [PMID 31289706](https://pubmed.ncbi.nlm.nih.gov/31289706/) | Observational prognostic study of postoperative atrial fibrillation and rehabilitation progress; no randomized rehabilitation comparison. |
| 27 | **Patel DK, Duncan MS, Shah AS, et al.** Association of Cardiac Rehabilitation With Decreased Hospitalization and Mortality Risk After Cardiac Valve Surgery. JAMA Cardiol. 2019;4(12):1250-1259.  [DOI: 10.1001/jamacardio.2019.4032](https://doi.org/10.1001/jamacardio.2019.4032) \| [PMID 31642866](https://pubmed.ncbi.nlm.nih.gov/31642866/) | Observational Medicare claims cohort; rehabilitation attendance was an observed exposure, not a randomized intervention. |
| 28 | **Shah RM, Hirji SA, Jolissaint JS, et al.** Comparison of Sex-Based Differences in Home or Nonhome Discharge Utilization of Rehabilitative Services and Outcomes Following Transcatheter Aortic Valve Implantation in the United States. Am J Cardiol. 2019;123(12):1983-1991.  [DOI: 10.1016/j.amjcard.2019.03.008](https://doi.org/10.1016/j.amjcard.2019.03.008) \| [PMID 30952379](https://pubmed.ncbi.nlm.nih.gov/30952379/) | Observational hospital-discharge database analysis of rehabilitative service use after TAVI; not a randomized trial. |
| 29 | **Song F, Zhan H, Liang Y, et al.** Cardiac rehabilitation improved oxygen uptake measured by cardiopulmonary exercise test in patients after aortic valve surgery. Rev Cardiovasc Med. 2019;20(1):47-52.  [DOI: 10.31083/j.rcm.2019.01.3183](https://doi.org/10.31083/j.rcm.2019.01.3183) \| [PMID 31184096](https://pubmed.ncbi.nlm.nih.gov/31184096/) | Retrospective comparison of exercise rehabilitation and usual care after aortic valve surgery; not a randomized trial. |
| 30 | **Tarro Genta F, Tidu M, Corbo P, et al.** Predictors of survival in patients undergoing cardiac rehabilitation after transcatheter aortic valve implantation. J Cardiovasc Med (Hagerstown). 2019;20(9):606-615.  [DOI: 10.2459/jcm.0000000000000829](https://doi.org/10.2459/jcm.0000000000000829) \| [PMID 31246699](https://pubmed.ncbi.nlm.nih.gov/31246699/) | Prognostic cohort of TAVI patients completing residential rehabilitation; no randomized control group. |
| 31 | **Bhattal GK, Park KE, Winchester DE.** Home-Based Cardiac Rehabilitation (HBCR) In Post-TAVR Patients: A Prospective, Single-Center, Cohort, Pilot Study. Cardiol Ther. 2020;9(2):541-548.  [DOI: 10.1007/s40119-020-00186-3](https://doi.org/10.1007/s40119-020-00186-3) \| [PMID 32535753](https://pubmed.ncbi.nlm.nih.gov/32535753/) | Single-arm home rehabilitation pilot after TAVR; pre/post assessments without a randomized comparator. |
| 32 | **Eichler S, Völler H, Reibis R, et al.** Geriatric or cardiac rehabilitation? Predictors of treatment pathways in advanced age patients after transcatheter aortic valve implantation. BMC Cardiovasc Disord. 2020;20(1):158.  [DOI: 10.1186/s12872-020-01452-x](https://doi.org/10.1186/s12872-020-01452-x) \| [PMID 32252646](https://pubmed.ncbi.nlm.nih.gov/32252646/) | Prospective cohort of rehabilitation referral pathways after TAVI; cardiac versus geriatric rehabilitation was selected clinically. |
| 33 | **Tabet JY, Meurin P, Ben Driss A, et al.** Early exercise training feasibility after aortic valve repair: A multicentre prospective French survey on behalf of the Aortic Valve repair International Registry (AVIATOR). Arch Cardiovasc Dis. 2020;113(3):168-175.  [DOI: 10.1016/j.acvd.2019.11.006](https://doi.org/10.1016/j.acvd.2019.11.006) \| [PMID 32067947](https://pubmed.ncbi.nlm.nih.gov/32067947/) | Prospective single-arm cohort with pre/post exercise assessments after aortic valve repair; no randomized comparator. |
| 34 | **Vendrik J, Vlastra W, van Mourik MS, et al.** Early mobilisation after transfemoral transcatheter aortic valve implantation: results of the MobiTAVI trial. Neth Heart J. 2020;28(5):240-248.  [DOI: 10.1007/s12471-020-01374-5](https://doi.org/10.1007/s12471-020-01374-5) \| [PMID 32112292](https://pubmed.ncbi.nlm.nih.gov/32112292/) | Nonrandomized allocation: early versus routine mobilization was determined by procedure completion time. |
| 35 | **Kim SH, Cha S, Kang S, et al.** High prevalence of physical inactivity after heart valve surgery and its association with long-term mortality: A nationwide cohort study. Eur J Prev Cardiol. 2021;28(7):749-757.  [DOI: 10.1177/2047487320903877](https://doi.org/10.1177/2047487320903877) \| [PMID 33611453](https://pubmed.ncbi.nlm.nih.gov/33611453/) | Observational national database cohort relating physical activity to mortality after valve surgery; no randomized exercise intervention. |
| 36 | **Kleczynski P, Trebacz J, Stapor M, et al.** Inpatient Cardiac Rehabilitation after Transcatheter Aortic Valve Replacement Is Associated with Improved Clinical Performance and Quality of Life. J Clin Med. 2021;10(10):2125.  [DOI: 10.3390/jcm10102125](https://doi.org/10.3390/jcm10102125) \| [PMID 34068973](https://pubmed.ncbi.nlm.nih.gov/34068973/) | Retrospective registry comparing rehabilitation recipients with eligible patients who declined the programme; no random allocation. |
| 37 | **Penati C, Incorvaia C, Mollo V, et al.** Cardiac rehabilitation outcome after transcatheter aortic valve implantation. Monaldi Arch Chest Dis. 2021;91(2):1621.  [DOI: 10.4081/monaldi.2021.1621](https://doi.org/10.4081/monaldi.2021.1621) \| [PMID 33849260](https://pubmed.ncbi.nlm.nih.gov/33849260/) | Observational pre/post rehabilitation study after TAVI; no randomized rehabilitation comparison. |
| 38 | **Yu Z, Zhao Q, Ye Y, et al.** Comprehensive Geriatric Assessment and Exercise Capacity in Cardiac Rehabilitation for Patients Referred to Transcatheter Aortic Valve Implantation. Am J Cardiol. 2021;158:98-103.  [DOI: 10.1016/j.amjcard.2021.07.045](https://doi.org/10.1016/j.amjcard.2021.07.045) \| [PMID 34465453](https://pubmed.ncbi.nlm.nih.gov/34465453/) | Retrospective analysis of patients receiving geriatric assessment and rehabilitation around TAVI; not a randomized trial. |
| 39 | **Borsoi L, Callea G, Tarricone R.** How to Reduce Inequity of Access to Cardiac Rehabilitation After Surgical Aortic Valve Replacement. Recommendations for the Post-COVID-19 Era From a Real-World, Population-Based Study. Clin Ther. 2022;44(4):491-507.  [DOI: 10.1016/j.clinthera.2022.02.002](https://doi.org/10.1016/j.clinthera.2022.02.002) \| [PMID 35249767](https://pubmed.ncbi.nlm.nih.gov/35249767/) | Retrospective population-based study of access to rehabilitation after surgical aortic valve replacement; not a randomized trial. |
| 40 | **Brocki BC, Andreasen JJ, Aaroe J, et al.** Exercise-Based Real-time Telerehabilitation for Older Adult Patients Recently Discharged After Transcatheter Aortic Valve Implantation: Mixed Methods Feasibility Study. JMIR Rehabil Assist Technol. 2022;9(2):e34819.  [DOI: 10.2196/34819](https://doi.org/10.2196/34819) \| [PMID 35471263](https://pubmed.ncbi.nlm.nih.gov/35471263/) | Prospective nonrandomized single-arm telerehabilitation feasibility study after TAVI. |
| 41 | **Guduguntla V, Yaser JM, Keteyian SJ, et al.** Variation in Cardiac Rehabilitation Participation During Aortic Valve Replacement Episodes of Care. Circ Cardiovasc Qual Outcomes. 2022;15(7):e009175.  [DOI: 10.1161/circoutcomes.122.009175](https://doi.org/10.1161/circoutcomes.122.009175) \| [PMID 35559710](https://pubmed.ncbi.nlm.nih.gov/35559710/) | Observational registry and claims analysis of rehabilitation participation after valve replacement; not a randomized trial. |
| 42 | **Jafri SH, Hushcha P, Dorbala P, et al.** Physical and Psychological Well-being Effects of Cardiac Rehabilitation on Patients Following Mitral Valve and Aortic Valve Procedures. J Cardiopulm Rehabil Prev. 2022;42(2):90-96.  [DOI: 10.1097/hcr.0000000000000609](https://doi.org/10.1097/hcr.0000000000000609) \| [PMID 34793360](https://pubmed.ncbi.nlm.nih.gov/34793360/) | Rehabilitation cohort comparing patients after mitral versus aortic valve procedures; no randomized rehabilitation comparison. |
| 43 | **Nusbickel AJ, Randall MH, Plasschaert JM, et al.** Cardiac Rehabilitation Referral After Transcatheter Aortic Valve Replacement. Crit Pathw Cardiol. 2022;21(4):162-164.  [DOI: 10.1097/hpc.0000000000000302](https://doi.org/10.1097/hpc.0000000000000302) \| [PMID 36413392](https://pubmed.ncbi.nlm.nih.gov/36413392/) | Nonrandomized before/after quality improvement project evaluating rehabilitation referral following TAVR. |
| 44 | **Schmidt T, Kowalski M, Bjarnason-Wehrens B, et al.** Feasibility of inpatient cardiac rehabilitation after percutaneous mitral valve reconstruction using clipping procedures: a retrospective analysis. BMC Sports Sci Med Rehabil. 2022;14(1):120.  [DOI: 10.1186/s13102-022-00517-y](https://doi.org/10.1186/s13102-022-00517-y) \| [PMID 35787297](https://pubmed.ncbi.nlm.nih.gov/35787297/) | Retrospective single-arm rehabilitation cohort after mitral valve clipping; no randomized comparator. |
| 45 | **Shimomoto Y, Mukaiyama K, Hori T, et al.** Postoperative delirium does not affect cardiopulmonary exercise testing in aged patients undergoing cardiac valve surgery. JA Clin Rep. 2022;8(1):64.  [DOI: 10.1186/s40981-022-00553-0](https://doi.org/10.1186/s40981-022-00553-0) \| [PMID 35962209](https://pubmed.ncbi.nlm.nih.gov/35962209/) | Retrospective comparison by postoperative delirium status; all patients received active exercise rehabilitation. |
| 46 | **Ashikaga K, Doi S, Yoneyama K, et al.** Efficacy and Safety of Home-Based Cardiac Telemonitoring Rehabilitation in Patients After Transcatheter Aortic Valve Implantation: Single-Center Usability and Feasibility Study. JMIR Rehabil Assist Technol. 2023;10:e45247.  [DOI: 10.2196/45247](https://doi.org/10.2196/45247) \| [PMID 37195764](https://pubmed.ncbi.nlm.nih.gov/37195764/) \| [Full text](https://pmc.ncbi.nlm.nih.gov/articles/PMC10233439/) | Nonrandomized feasibility study comparing home telerehabilitation with a historical outpatient rehabilitation cohort. |
| 47 | **Gempel S, Cohen M, Milian E, et al.** Inspiratory Muscle and Functional Performance of Patients Entering Cardiac Rehabilitation after Cardiac Valve Replacement. J Cardiovasc Dev Dis. 2023;10(4):142.  [DOI: 10.3390/jcdd10040142](https://doi.org/10.3390/jcdd10040142) \| [PMID 37103021](https://pubmed.ncbi.nlm.nih.gov/37103021/) | Retrospective chart review of inspiratory muscle and functional measures at rehabilitation entry; no randomized intervention. |
| 48 | **Ishihara K, Izawa KP, Morikawa T, et al.** Efficacy of MitraClip and continued multidisciplinary cardiac rehabilitation in preventing readmission of an older heart failure patient with severe multivalvular disease: A case report. J Cardiol Cases. 2023;27(1):23-26.  [DOI: 10.1016/j.jccase.2022.09.007](https://doi.org/10.1016/j.jccase.2022.09.007) \| [PMID 36618845](https://pubmed.ncbi.nlm.nih.gov/36618845/) | Single-patient case report combining MitraClip treatment and multidisciplinary rehabilitation. |
| 49 | **Sukul D, Albright J, Thompson MP, et al.** Predictors and Variation in Cardiac Rehabilitation Participation After Transcatheter Aortic Valve Replacement. JACC Adv. 2023;2(8):100581.  [DOI: 10.1016/j.jacadv.2023.100581](https://doi.org/10.1016/j.jacadv.2023.100581) \| [PMID 38938330](https://pubmed.ncbi.nlm.nih.gov/38938330/) | Observational linkage of TAVR registry and claims data to examine rehabilitation participation; not a randomized trial. |
| 50 | **Tarro Genta F, Marcassa C, Ceresa M, et al.** Predictors of survival in patients undergoing cardiac rehabilitation after transcatheter aortic valve replacement (TAVR): a multicenter retrospective study. Panminerva Med. 2023;65(2):220-226.  [DOI: 10.23736/s0031-0808.22.04444-5](https://doi.org/10.23736/s0031-0808.22.04444-5) \| [PMID 35315992](https://pubmed.ncbi.nlm.nih.gov/35315992/) | Retrospective prognostic study of survival among TAVR rehabilitation participants; no randomized comparator. |
| 51 | **Bai Z, Jiang Y, Wang M.** Effects of Phase Ⅰ Cardiac Rehabilitation Combined with Cognitive Behavioural Therapy on Cardiac Function, Exercise Capacity and Mental Health in Patients after Aortic Valve Replacement: A Retrospective Study. Heart Surg Forum. 2024;27(1):E048-E057.  [DOI: 10.59958/hsf.7103](https://doi.org/10.59958/hsf.7103) \| [PMID 38286640](https://pubmed.ncbi.nlm.nih.gov/38286640/) | Retrospective comparison of rehabilitation with or without cognitive behavioural therapy after aortic valve replacement. |
| 52 | **Gopal A, Detroyer I, de Alcantara Lima N, et al.** Improved Cardiac Rehabilitation Referral Rates following Coronary Artery Bypass Graft and Heart Valve Replacement in an Inner-City Veteran Population. Am J Med Qual. 2024;39(6):280-284.  [DOI: 10.1097/jmq.0000000000000206](https://doi.org/10.1097/jmq.0000000000000206) \| [PMID 39472764](https://pubmed.ncbi.nlm.nih.gov/39472764/) | Nonrandomized before/after quality improvement study of rehabilitation referral after cardiac surgery. |
| 53 | **Lauck SB, Yu M, Bancroft C, et al.** Early mobilization after transcatheter aortic valve implantation: observational cohort study. Eur J Cardiovasc Nurs. 2024;23(3):296-304.  [DOI: 10.1093/eurjcn/zvad081](https://doi.org/10.1093/eurjcn/zvad081) \| [PMID 37610363](https://pubmed.ncbi.nlm.nih.gov/37610363/) | Prospective observational cohort evaluating a nurse-led mobilization protocol; not a randomized trial. |
| 54 | **Tsujimoto K, Watanabe S, Kondo T, et al.** Effects of Early Gait Training on Inpatient Frailty After Transcatheter Aortic Valve Implantation. Cureus. 2024;16(6):e63086.  [DOI: 10.7759/cureus.63086](https://doi.org/10.7759/cureus.63086) \| [PMID 39055434](https://pubmed.ncbi.nlm.nih.gov/39055434/) | Retrospective record-based comparison by observed gait-training start date; no random allocation. |
| 55 | **Corovic M, Mosleh K, Karia E, et al.** JUMPSTART pilot: assessing the acceptability and feasibility of a novel early mobilization program following transcatheter aortic valve replacement. Front Cardiovasc Med. 2025;12:1568844.  [DOI: 10.3389/fcvm.2025.1568844](https://doi.org/10.3389/fcvm.2025.1568844) \| [PMID 40636829](https://pubmed.ncbi.nlm.nih.gov/40636829/) | Nonrandomized JUMPSTART pilot using post-discharge surveys of programme uptake and acceptability. |
| 56 | **Corovic M, Mosleh K, Puglisi O, et al.** JUMPSTART: evaluation of an early mobilization program following transcatheter aortic valve replacement. BMC Cardiovasc Disord. 2025;25(1):216.  [DOI: 10.1186/s12872-025-04665-0](https://doi.org/10.1186/s12872-025-04665-0) \| [PMID 40133799](https://pubmed.ncbi.nlm.nih.gov/40133799/) | Prospective observational JUMPSTART evaluation; programme participation was not randomized. |
| 57 | **Katano S, Kono Y, Yano T, et al.** Impact of in-hospital cardiac rehabilitation on hospital-associated disability after transcatheter aortic valve implantation. J Am Geriatr Soc. 2025;73(2):506-519.  [DOI: 10.1111/jgs.19265](https://doi.org/10.1111/jgs.19265) \| [PMID 39570021](https://pubmed.ncbi.nlm.nih.gov/39570021/) | Retrospective national database cohort comparing in-hospital rehabilitation participation after TAVI; not a randomized trial. |
| 58 | **Kim Y, Song SW, Lee H, et al.** Functional Benefits of Inpatient Cardiac Rehabilitation After Open Aortic and Valvular Surgery: A Retrospective Cohort Study. Healthcare (Basel). 2025;13(15):1816.  [DOI: 10.3390/healthcare13151816](https://doi.org/10.3390/healthcare13151816) \| [PMID 40805849](https://pubmed.ncbi.nlm.nih.gov/40805849/) | Retrospective single-arm inpatient rehabilitation cohort after aortic or valve surgery; no randomized comparator. |
| 59 | **Malvindi PG, Ceravolo MG, Capecci M, et al.** Telemedicine/Telerehabilitation to Expand Enhanced Recovery After Surgery Interventions in Minimally Invasive Mitral Valve Surgery. J Clin Med. 2025;14(3):750.  [DOI: 10.3390/jcm14030750](https://doi.org/10.3390/jcm14030750) \| [PMID 39941421](https://pubmed.ncbi.nlm.nih.gov/39941421/) | Descriptive programme-development report; the article contains no patient outcome data or randomized comparison. |
| 60 | **Ochiai K, Kameda I, Kato Y, et al.** Impact of Delayed Postoperative Rehabilitation on Patients with Valvular Heart Disease: A Retrospective Cohort Study. Prog Rehabil Med. 2025;10:20250006.  [DOI: 10.2490/prm.20250006](https://doi.org/10.2490/prm.20250006) \| [PMID 39989475](https://pubmed.ncbi.nlm.nih.gov/39989475/) | Retrospective database cohort comparing observed rehabilitation start times after valve surgery; no random allocation. |
| 61 | **Ohnishi Y, Yokote T, Kawamitsu K, et al.** Association Between Preoperative Short Physical Performance Battery and Hospitalization-Associated Disability After Transcatheter Aortic Valve Implantation. Cureus. 2025;17(8):e89977.  [DOI: 10.7759/cureus.89977](https://doi.org/10.7759/cureus.89977) \| [PMID 40951221](https://pubmed.ncbi.nlm.nih.gov/40951221/) | Retrospective prognostic cohort examining preoperative physical function and postoperative disability; all TAVI patients received rehabilitation. |
| 62 | **Wilder EH, Dellise NR, Dhaliwal G, et al.** Transcatheter Heart Valve Postprocedural Care: Improving the Cardiac Rehabilitation Referral Process Before Discharge. JACC Case Rep. 2025;30(36):105898.  [DOI: 10.1016/j.jaccas.2025.105898](https://doi.org/10.1016/j.jaccas.2025.105898) \| [PMID 41238265](https://pubmed.ncbi.nlm.nih.gov/41238265/) | Nonrandomized quality improvement project assessing rehabilitation referral documentation after transcatheter valve procedures. |
| 63 | **Braver J, Marwick TH, Nolan J, et al.** Cardiac rehabilitation after transcatheter aortic valve implantation before, during and after the COVID-19 pandemic: a whole-population study. Heart. 2026:heartjnl-2025-327186.  [DOI: 10.1136/heartjnl-2025-327186](https://doi.org/10.1136/heartjnl-2025-327186) \| [PMID 41932822](https://pubmed.ncbi.nlm.nih.gov/41932822/) | Retrospective national electronic-record cohort comparing rehabilitation participation after TAVI; not a randomized trial. |
| 64 | **Kobayashi D, Saitoh M, Hori K, et al.** Effect of Comprehensive Outpatient Cardiac Rehabilitation on All-Cause Mortality After Transcatheter Aortic Valve Implantation. Circ Rep. 2026;8(5):719-729.  [DOI: 10.1253/circrep.cr-25-0341](https://doi.org/10.1253/circrep.cr-25-0341) \| [PMID 42110866](https://pubmed.ncbi.nlm.nih.gov/42110866/) | Retrospective cohort comparing outpatient rehabilitation attenders and nonattenders after TAVI; no random allocation. |
| 65 | **Myszenski AL, Childers K, Seifferlein K, et al.** Hospital-Based Rehabilitation, Functional Ambulation Status at Discharge, and Hospital Outcomes Across Society of Thoracic Surgeons Risk Groups in Adult Patients After Transcatheter Aortic Valve Replacements. Cureus. 2026;18(2):e102918.  [DOI: 10.7759/cureus.102918](https://doi.org/10.7759/cureus.102918) \| [PMID 41798407](https://pubmed.ncbi.nlm.nih.gov/41798407/) | Retrospective TAVR cohort stratified by surgical risk and observed rehabilitation use; not a randomized trial. |
| 66 | **Müller M, Hanssen TA, Johansen D, et al.** Feasibility of early interval training in patients recovering from heart valve surgery due to infective endocarditis. Pilot Feasibility Stud. 2026;12(1):93.  [DOI: 10.1186/s40814-026-01830-w](https://doi.org/10.1186/s40814-026-01830-w) \| [PMID 42116217](https://pubmed.ncbi.nlm.nih.gov/42116217/) | Single-arm feasibility study of early interval training after valve surgery for endocarditis; no randomized comparator. |
| 67 | **Ohta K, Saito H, Shimizu M, et al.** Recovery of Physical Function in a Patient with Intensive Care Unit-acquired Weakness after Valvular Surgery: A Case Report. Phys Ther Res. 2026;29(1):66-70.  [DOI: 10.1298/ptr.25-e10366](https://doi.org/10.1298/ptr.25-e10366) \| [PMID 42094787](https://pubmed.ncbi.nlm.nih.gov/42094787/) | Single-patient case report of rehabilitation for intensive care unit-acquired weakness after valve surgery. |
| 68 | **Stamate TC, Gavril RS, Roca M, et al.** Systemic Inflammatory Burden Is Independently Associated with Anemia After Transcatheter Aortic Valve Implantation in Patients Referred to Cardiac Rehabilitation. Biomedicines. 2026;14(7):1584.  [DOI: 10.3390/biomedicines14071584](https://doi.org/10.3390/biomedicines14071584) \| [PMID 42512056](https://pubmed.ncbi.nlm.nih.gov/42512056/) | Retrospective observational comparison by anaemia status at rehabilitation entry after TAVI; no randomized rehabilitation intervention. |
| 69 | **Takegawa K, Iwanaga Y, Kanaoka K, et al.** Outpatient Cardiac Rehabilitation and Long-Term Clinical Outcomes After Transcatheter Aortic Valve Replacement. J Am Geriatr Soc. 2026;74(4):1071-1079.  [DOI: 10.1111/jgs.70356](https://doi.org/10.1111/jgs.70356) \| [PMID 41714308](https://pubmed.ncbi.nlm.nih.gov/41714308/) | Observational national claims cohort using propensity matching to compare outpatient rehabilitation participation after TAVR. |
| 70 | **Tashiro N, Matsumoto Y, Abe S, et al.** Impact of Geographic Factors on Outpatient Cardiac Rehabilitation Adherence After Transcatheter Aortic Valve Implantation. Circ Rep. 2026;8(6):884-890.  [DOI: 10.1253/circrep.cr-25-0290](https://doi.org/10.1253/circrep.cr-25-0290) \| [PMID 42273319](https://pubmed.ncbi.nlm.nih.gov/42273319/) | Retrospective cohort examining geographical predictors of rehabilitation attendance after TAVI; no randomized comparison. |

# Appendix 4 Study populations and baseline characteristics

## Table S3 Demographic and procedural characteristics

| **Study** | **Country** | **Procedure** | **Valve disease or indication** | **Sample size T/C** | **Age T/C years** | **BMI T/C kg/m²** | **Female n/N T/C** |
| --- | --- | --- | --- | --- | --- | --- | --- |
| Pressler 2016 | Germany | TAVR/TAVI | Severe aortic stenosis | 15/15 | 81 ± 7/81 ± 5 | 26.9 ± 3.1/26.9 ± 3.7 | 6/13 / 6/14 |
| Cargnin 2019 | Brazil | Surgery | Aortic or mitral valve disease | 13 / 12 | 62.8 ± 8.8/60.1 ± 12.5 | 26.7 ± 1.7/28.1 ± 1.4 | 4/13 / 6/12 |
| Nilsson 2018 | Sweden | Surgery | Aortic stenosis | 6 / 6 | 58.5 (39–75)/65.5 (60–71) | NR | 1/6 / 2/6 |
| Rogers 2018 | United Kingdom | TAVR/TAVI | Severe symptomatic aortic stenosis | 13 / 14 | 82.92 ± 6.0/81.21 ± 3.6 | 27.24 ± 3.6/28.13 ± 4.9 | 7/13 / 8/14 |
| Sire 1987 | Norway | Surgery | Aortic stenosis, regurgitation, or mixed disease | 21 / 23 | 45.5 ± 11.7/45.5 ± 12.2 | NR | 3/21 / 5/23 |
| Cao 2013 | China | Surgery | Valvular heart disease | 50 / 47 | 48.5 ± 8.4/49.2 ± 13.5 | NR | NR |
| Li 2020 | China | Surgery | Severe valvular heart disease | 40 / 40 | 56.34 ± 2.52/56.15 ± 2.62 | 23.97 ± 2.42/24.21 ± 2.52 | 18/40 / 17/40 |
| Lin 2004 | China | Surgery | Rheumatic valve disease | 55/49 | 32.8 ± 12.1/29.8 ± 9.4 | NR | 24/55 / 21/49 |
| Sun 2021 | China | Surgery | Severe valvular heart disease | 100 / 100 | 69.1 ± 2.7/70.1 ± 3.8 | 24.1 ± 2.7/25.1 ± 2.3 | 46/100 / 44/100 |
| Zou 2020 | China | Surgery | Rheumatic heart disease | 40 / 40 | 53.21 ± 2.68/53.78 ± 2.91 | NR | 11/40 / 13/40 |
| Tao 2020 | China | Surgery | Mitral stenosis/regurgitation or tricuspid regurgitation | 35 / 35 | 82.32 ± 1.26/82.34 ± 1.14 | NR | 17/35 / 19/35 |
| Tamulevičiūtė-Prascienė 2021 | Lithuania | Surgery | Aortic, mitral or tricuspid valve disease | 60 / 56 | 75.9 ± 6.6/76.4 ± 6.6 | 27.9 ± 3.9/27.6 ± 4.9 | 30/60 / 28/56 |
| Weber 2021 | Germany | TAVR/TAVI | Severe symptomatic aortic stenosis; high surgical risk | 58 / 50 | 82.2 ± 5.8/81.7 ± 5.0 | 26.3 ± 4.7/27.4 ± 4.5 | 31/58 / 24/50 |
| Xue 2022 | China | Surgery | Valvular heart disease; TAVI excluded | 44 / 43 | 58.20 ± 5.27/57.98 ± 4.18 | 23.09 ± 2.23/23.28 ± 2.59 | 15/44 / 16/43 |
| Xu 2022 | China | TAVR/TAVI | Aortic stenosis, regurgitation or combined lesions | 48 / 48 | 72.01 ± 5.56/72.45 ± 7.68 | 22.72 ± 3.72/22.65 ± 3.17 | 23/48 / 19/48 |
| Hu (ENERGY) 2023 | China | TAVR/TAVI | Aortic stenosis | 33/33 | 70.36 ± 6.93/70.94 ± 6.35 | 23.96 ± 3.78/24.29 ± 3.31 | 15/33 / 15/33 |
| Vitez 2023 | Slovenia | TAVR/TAVI | Aortic stenosis | 20/19 | 81 ± 5/82 ± 4 | 30.9 ± 4.9/26.3 ± 5.2 | 7/10 / 7/13 |
| Song 2024 | China | TAVR/TAVI | Aortic stenosis with chronic heart failure | 30 / 30 | 76.8 ± 3.4/75.6 ± 3.5 | 23.9 ± 3.9/23.8 ± 2.3 | 10/30 / 12/30 |
| Shi 2025 | United States | TAVR/TAVI | Aortic valve disease treated by TAVR | 15/18 | 85.1 ± 9.1/84.9 ± 5.1 | 27.5 ± 6.4/27.9 ± 4.8 | 1/15 / 9/18 |
| Gao 2025 | China | Surgery | Valvular disease with atrial fibrillation | 95/95 | 56.27 ± 6.63/54.91 ± 9.87 | 22.06 ± 3.30/23.18 ± 3.04 | 54/89 / 45/85 |
| Liu 2025 | China | Surgery | Mitral and/or aortic valve disease | 492/486 | 52 ± 11.6/51 ± 12.3 | 23.4 ± 3.5/23.3 ± 3.5 | 212/399 / 200/419 |
| Li 2023 | China | Surgery | Valvular heart disease | 46 / 46 | 59.30 ± 3.27/58.66 ± 3.49 | NR | 20/46 / 21/46 |
| Gao 2024 | China | Surgery | Valvular heart disease | 40 / 40 | NR | NR | 16/40 / 19/40 |
| Zhang 2026 | China | Surgery | Valvular heart disease | 40 / 40 | 48.73 ± 3.97/48.62 ± 3.58 | NR | 17/40 / 18/40 |
| Sibilitz 2016 | Denmark | Surgery | Aortic, mitral, tricuspid or pulmonary valve disease | 72 / 75 | 62.0 ± 11.5/61.0 ± 9.9 | 26.2 ± 4.2/26.1 ± 3.9 | 13/72 / 22/75 |
| Luo 2023 | China | Surgery/TAVR | Valvular heart disease; NYHA II–III | 82 / 83 | 63.48 ± 10.90/63.34 ± 9.61 | 23.98 ± 3.67/23.35 ± 3.28 | 37/82 / 39/83 |
| Yu 2025 | China | Surgery | Valvular heart disease; NYHA II–III | 76/76 | 54 ± 9/55 ± 9 | 24.5 ± 3.8/24.6 ± 4.1 | 27/76 / 33/76 |
| Ahmad 2024 | Egypt | Surgery | Valvular heart disease | 19/19 | 33.44 ± 5.77/33.20 ± 6.29 | 28.11 ± 6.41/29.83 ± 8.36 | 10/16 / 11/15 |
| Atef 2026 | Egypt | Surgery | Rheumatic heart disease | 20 / 20 | 26.85 ± 3.66/28.30 ± 3.20 | 26.27 ± 3.98/26.31 ± 5.07 | NR |

Abbreviations: T/C, intervention/control; BMI, body mass index; NYHA, New York Heart Association; NR, not reported or not ascertainable; TAVR/TAVI, transcatheter aortic valve replacement/implantation; n/N, number of women/number assessed.

## Table S4 Baseline cardiac function and functional status

| **Study** | **NYHA or functional class** | **LVEF %** | **6MWD m** | **Peak VO₂** | **Frailty or functional proxy** | **Baseline assessment anchor** |
| --- | --- | --- | --- | --- | --- | --- |
| Pressler 2016 | I/II/III: 1/10/2 / 4/6/4 | 58 ± 8 / 57 ± 10 | 366 ± 93 / 319 ± 104 | 12.7 ± 3.0 / 15.0 ± 3.3 mL/kg/min | No formal score. Cane/walker use reported. | Before training, approximately 3 months after TAVI |
| Cargnin 2019 | Median (range): 2 (1–4) / 2 (1–4) | 59.5 ± 2.9 / 60.1 ± 4.8 | 411.4 ± 103.2 / 324.4 ± 135.9 | NR | NR | Week before surgery |
| Nilsson 2018 | NR | NR | NR | 22 (17–35) / 20 (15–27), median (range), mL/kg/min | NR | 5–6 weeks postoperatively |
| Rogers 2018 | NR | ≥50%: 9/13 / 12/14; 30–49%: 3/13 / 2/14; <30%: 1/13 / 0/14 | 325.2 ± 22.8 / 401.0 ± 41 | NR | Fried frail: 3/13 / 2/12 assessed; Edmonton 5.08 ± 2.2 / 5.25 ± 1.8 | 30 days post-TAVI, before randomization |
| Sire 1987 | NR | NR | NR | NR | NR | 2 months after surgery |
| Cao 2013 | NR | NR | NR | NR | NR | Peri-surgical baseline; exact assessment day NR |
| Li 2020 | I–III reported; T counts internally inconsistent | 45.81 ± 6.73 / 46.48 ± 6.24 | 328.14 ± 50.82 / 322.24 ± 51.56 | NR | NR | Preoperative |
| Lin 2004 | II/III/IV: 7/38/10 / 8/33/8 | NR | NR | NR | NR | Preoperative |
| Sun 2021 | NR | NR | NR | NR | NR | Admission before surgery |
| Zou 2020 | NR | 50.45 ± 1.21 / 50.33 ± 1.22 | NR | NR | NR | Before nursing intervention; exact day NR |
| Tao 2020 | II/III/IV: 20/12/3 / 19/14/2 | NR | NR | NR | NR | Preoperative NYHA |
| Tamulevičiūtė-Prascienė 2021 | II/III: 25/35 / 20/36 | 46.9 ± 8.6 / 44.9 ± 9.4 | 246 ± 93.5 / 231 ± 98.8 | 11.2 ± 4.38 / 11.3 ± 2.81 mL/kg/min | SPPB-defined frail: 20/53 / 15/42 assessed; gait-speed frail also reported | CR admission, about 2 weeks after procedure |
| Weber 2021 | Mean 3.1 ± 0.5 / 3.1 ± 0.4; >II: 53/58 / 47/50 | 53.5 ± 11.9 / 54.6 ± 14.1 | 221.4 ± 109.1 / 223.1 ± 124.5 | NR | NR | Pre-TAVR inclusion |
| Xue 2022 | NR | NR | 311.52 ± 54.98 / 309.58 ± 57.44 | NR | SPPB: 8.86 ± 1.52 / 8.74 ± 1.83; no categorical frailty score | Day before surgery for physical tests; Table 1 LVEF is labelled postoperative |
| Xu 2022 | I/II/III/IV: 5/17/23/3 / 3/17/25/3 | 63.00 (50.00–70.00) / 60.00 (49.00–68.00), median (IQR) | 340.81 ± 86.64 / 377.67 ± 84.09 | 18.72 (15.57–21.94) / 17.75 (15.38–22.83), median (IQR), mL/kg/min | Robust/pre-frail/frail: 18/24/6 / 24/23/1 (counts; percentages contain source typos) | At baseline assessment around admission; exact relation to procedure for each test NR |
| Hu (ENERGY) 2023 | I/II/III: 18/15/0 / 17/15/1 | 53.30 ± 10.75 / 57.70 ± 9.49 | 385.06 ± 70.62 / 377.03 ± 62.07 | 11.15 ± 2.92 / 11.40 ± 3.36 mL/kg/min | NR | Before training, median 157 days after TAVR |
| Vitez 2023 | I/II/III: 1/8/1 / 0/13/0 | 57 ± 13 / 55 ± 13 | 346 ± 77 / 313 ± 68 | 0.87 (0.66–0.98) / 0.76 (0.64–0.91), median (IQR) | NR | Before study training, 3–6 months after TAVI |
| Song 2024 | II–III required; arm-specific distribution NR | 55.0 ± 6.6 / 52.6 ± 7.4 | 293.9 ± 37.6 / 295.3 ± 54.7 | 13.9 ± 2.7 / 14.1 ± 2.8 mL/kg/min | NR | Before CR after TAVR; exact interval inconsistent |
| Shi 2025 | III–IV 8/18 / 9/14 | 60.6 ± 9.4 / 57.0 ± 8.1 | NR | NR | Frailty index A/B/C: 0.20 ± 0.09 / 0.23 ± 0.09 / 0.24 ± 0.08 | In hospital after TAVR, before home programme |
| Gao 2025 | Functional classes I/II/III/IV: 4/5/53/27 / 2/4/51/28 (as reported) | NR | NR | NR | NR | Preoperative; demographic table uses analysed cohort |
| Liu 2025 | NR | <50%: 32/399 / 29/419 | NR | 16.4 (13.4–20.6) / 16.6 (13.6–20.8), median (IQR), mL/kg/min | Handgrip proxy: 28.4 (21.6–38.2) / 28.6 (22.3–37.7) kg, median (IQR); no total frailty score in main PDF | Preoperative |
| Li 2023 | NR | 39.05 ± 4.23 / 38.72 ± 4.05 | 239.13 ± 37.70 / 251.04 ± 34.29 | NR | NR | Before postoperative nursing intervention; exact day NR |
| Gao 2024 | II/III/IV: 13/22/5 / 11/21/8 | 53.69 ± 4.52 / 53.58 ± 4.35 | 100.98 ± 19.02 / 100.87 ± 18.96 | NR | NR | Before nursing intervention; exact postoperative day NR |
| Zhang 2026 | NR | 40.72 ± 5.91 / 40.65 ± 5.88 | 71.75 ± 14.69 / 72.28 ± 14.78 | NR | NR | Before nursing intervention; exact postoperative day NR |
| Sibilitz 2016 | I–II / III–IV: 53/19 / 52/23 | 55 ± 9.6 / 54 ± 10.2 | 546.8 / 542.9 (model-estimated means at 1 month; SD NR) | 21.0 / 21.1 (back-transformed model means at 1 month; SD NR), mL/kg/min | NR | Clinical baseline before randomization; functional testing at 1 month before training |
| Luo 2023 | II/III: 45/37 / 29/54 | 57.55 ± 11.11 / 55.73 ± 14.68 | NR | NR | NR | Preoperative admission |
| Yu 2025 | II/III A/B: 35/41 / 40/36 | 58 ± 7 / 58 ± 7 | 370 ± 70 / 384 ± 88 | NR | NR | Preoperative admission |
| Ahmad 2024 | NR | NR | 407.50 ± 52.38 / 339.13 ± 60.82 | NR | NR | Preoperative testing; demographic table describes completers |
| Atef 2026 | NR | NR | 252.00 ± 43.99 / 246.25 ± 48.64 | NR | NR | Within 48 h postoperatively before intervention |

# Appendix 5 Intervention and comparator details

## Table S5 Intervention components and comparator care

| **Study** | **Intervention components** | **Comparator components** | **Shared background care** | **Additional nonexercise components** | **Comparison family** |
| --- | --- | --- | --- | --- | --- |
| Pressler 2016 | Cycle ergometry followed by progressive machine-based resistance exercise. | No structured exercise programme. Ongoing care by family physicians and cardiologists. | Usual medical care | None specified | Usual care / advice |
| Cargnin 2019 | Pressure-threshold IMT with diaphragmatic breathing; weekly load adjustment. | Identical device and schedule at the minimum device load, plus the same postoperative routine care. | Usual medical care | None specified | Sham IMT |
| Nilsson 2018 | Heart-rate-guided cycle ergometry, 36 sessions. | General activity recommendations (≥30 min light/moderate activity ≥5 days/week or ≥20 min vigorous activity ≥3 days/week), with three encouragement contacts. | Standard early postoperative mobilization and discharge advice in both groups. | None specified | Usual care / advice |
| Rogers 2018 | Individualized cardiovascular and resistance/functional training after biopsychosocial assessment. | Routine outpatient follow-up, drug treatment and comorbidity management; general early-mobilization advice, without formal CR. | Usual medical care | Biopsychosocial assessment; individualized goal setting. | Usual care / advice |
| Sire 1987 | Cycling warm-up, dynamic/isometric limb exercises, calisthenics, volleyball, home exercise and vocational rehabilitation. | Not encouraged to undertake systematic training; habitual daily activity described as moderate. | Usual medical care | Vocational assessment and return-to-work assistance. | Usual care / advice |
| Cao 2013 | Seven-step cardiac rehabilitation, activities-of-daily-living training and serial health education. | Conventional nursing after valve surgery; exercise dose not described. | Usual medical care | Health education and self-care training. | Usual care / advice |
| Li 2020 | 6MWT-guided MOTOmed exercise for lower-capacity patients or aerobic/muscle exercise for higher-capacity patients, plus motivational interviewing. | Preoperative education, lifestyle and diet advice, monitoring and routine preparation. | Usual medical care | Motivational interviewing in intervention arm. | Usual care / advice |
| Lin 2004 | Preoperative breathing/cough training and limb range-of-motion; postoperative graded mobilization; individualized aerobic and strength exercise at home. | Conventional preoperative medical treatment, valve surgery, postoperative monitoring, ventilation, analgesia, cardiovascular drugs, antibiotics and anticoagulation. No structured rehabilitation described. | Usual medical care | Preoperative psychological support and video education. | Usual care / advice |
| Sun 2021 | 6MWT-based exercise selection: device-assisted training for grades 1–2, aerobic and muscle exercises for grades 3–4. | Education, lifestyle advice, monitoring and preoperative preparation. | Usual medical care | None specified | Usual care / advice |
| Zou 2020 | Abdominal breathing, passive and active limb exercise, standing and slow walking, with psychological and lifestyle support. | Routine postoperative nursing; detailed exercise content NR. | Usual medical care | Psychological support, diet/lifestyle guidance and individualized rehabilitation planning. | Usual care / advice |
| Tao 2020 | Passive-to-active limb movements, abdominal breathing, simulated cycling and progressive corridor walking. | Symptomatic treatment, airway care and prevention of severe complications; structured exercise NR. | Usual medical care | None specified | Usual care / advice |
| Tamulevičiūtė-Prascienė 2021 | Additional lower-body resistance and balance sessions on top of an identical multidisciplinary inpatient CR programme. | 20-day CR with cycling 6/week (30–50% peak watts or 60–70% HRmax), gymnastics 30 min 5/week, respiratory exercise 15 min daily, education, diet counselling and psychological support. | Full exercise-based multidisciplinary CR in both groups. | Only T received fortnightly telephone follow-up and specific home-training encouragement. | Active rehabilitation add-on |
| Weber 2021 | Pre-TAVR IMT and walking plus twice-daily postoperative mobilization/physiotherapy. | No preventive pre-TAVR physiotherapy. Postoperative physiotherapy 30 min once daily until discharge. | Usual medical care | None specified | Active rehabilitation add-on |
| Xue 2022 | Four-day progression from bed exercises to sitting, standing and ward walking, followed by walking up to 500 m and stationary cycling. | Routine clinical care during admission; no rehabilitation programme. | Usual medical care | None specified | Usual care / advice |
| Xu 2022 | Progressive threshold IMT on top of usual exercise-based multidisciplinary CR. | Sham device with permanently open valve (<1 cmH2O) on the same schedule. Both arms receive mobilization, sit-to-stand and walking, 30 min once daily 3–5 days/week, plus education/diet/psychological care. | Usual CR in both arms; Borg 11–12 for common physical exercise. | None specified | Sham IMT |
| Hu (ENERGY) 2023 | CPET-guided moderate-intensity continuous aerobic training with progressive duration. | One-time guideline-based advice on physical activity, without the structured study programme. | Usual medical care | None specified | Usual care / advice |
| Vitez 2023 | Supervised centre-based cycling, resistance and balance training. | 30-min physiotherapist education plus prescribed home exercise ≥150 min/week, 20–45 min/session at 40–75% heart-rate reserve, with monthly review. | Usual medical care | None specified | Active exercise strategy comparison |
| Song 2024 | Individualized progressive aerobic/cycle training within holistic CR. | Conventional rehabilitation/lifestyle management without individualized exercise. Psychological-care content is inconsistently described. | Medical, dietary, educational and risk-factor management described as common CR components. | None specified | Usual care / advice |
| Shi 2025 | Home balance/flexibility/strength/endurance training plus cognitive-behavioural intervention | 30-min weekly telephone education on exercise/diet; walking 30 min/day or as tolerated. Not a no-activity control. | Usual medical care | Arm A adds CBI, diary and adherence incentive; arm B does not. | Usual care / advice |
| Gao 2025 | Earlier progressive mobilization, respiratory physiotherapy, and structured pain/nutrition/psychological management. | Preoperative breathing/cough training and education; POD 1 supine rest, POD 2–4 sitting/cough care, ambulation after device removal around POD 6; conventional reactive analgesia. | Shared preoperative respiratory exercises, education and preparation. | Analgesia, nutritional management, psychological support and monitoring also differ. | Active rehabilitation add-on |
| Liu 2025 | Physiotherapist-led education, progressive IMT, active-cycle breathing techniques and early mobilization. | Nurse teaches deep breathing, cough exercise and incentive spirometry on admission; patients continue these autonomously without supervision throughout admission. | Usual medical care | None specified | Active rehabilitation add-on |
| Li 2023 | Diaphragmatic/pursed-lip breathing and incentive training; passive-to-active limb exercise, ADL, walking/stairs, resistance and aerobic activities. | Routine postoperative nursing, medication/complication monitoring, education, psychological support and simple limb movements. | Usual medical care | None specified | Usual care / advice |
| Gao 2024 | Video-guided passive/active movements, simulated cycling and progressive walking, plus cognitive-behavioural psychological care. | Routine postoperative care plus guided early basic upper-limb abduction, flexion/extension and external-rotation exercises. | Usual medical care | CBT-related education, relaxation/meditation, hypnosis and music therapy in intervention arm. | Active rehabilitation add-on |
| Zhang 2026 | Passive movements within 12 h, active bed activity on POD 1–2, standing/walking on POD 3–5, breathing/coughing and psychological support. | Vital-sign/drain care, dietary management and complication prevention; no structured exercise described. | Usual medical care | Structured psychological support and education. | Usual care / advice |
| Sibilitz 2016 | Graduated aerobic exercise and lower-body strength training, plus five nurse-led psycho-educational consultations. | Guideline-based care and physician review at 1 and 4 months, without assigned structured exercise or psychoeducation. | Early postoperative mobilization in both groups before structured study exercise. | Nurse-led psychosocial/disease-coping consultations. | Usual care / advice |
| Luo 2023 | TIME bundle: intensive IMT, symptom-limited corridor walking and physiotherapist-led education. | Routine preoperative nursing education and booklet; no additional prehabilitation bundle. | Standard preoperative nursing education, medical care and postoperative physiotherapy. | Additional PT education on respiratory techniques, activity and postoperative self-management. | Usual care / advice |
| Yu 2025 | Progressive tapering-flow-resistance IMT | Sham arm receives identical device/schedule at 10% MIP | Admission education, nursing, medical team and postoperative physiotherapy. | None specified | Two contrasts: sham IMT; usual care |
| Ahmad 2024 | Supervised bedside lower-limb mini-bike cycling added to the common inpatient cardiac rehabilitation programme. | Daily physiotherapist-supervised inpatient CR: breathing/coughing, active limb movements, early sitting/standing, corridor walking and stair progression. | Same inpatient CR, sternal precautions and medication in both arms. | None specified | Active rehabilitation add-on |
| Atef 2026 | Six resistance exercises added to identical moderate-intensity continuous aerobic training. | One-time guideline-based advice on physical activity | Usual medical care | None specified | Usual care / advice |

Abbreviations: T/C, intervention/control; ADL, activities of daily living; CBI, cognitive-behavioural intervention; CBT, cognitive-behavioural therapy; CPET, cardiopulmonary exercise testing; CR, cardiac rehabilitation; cmH2O, centimetres of water; HRmax, maximum heart rate; IMT, inspiratory muscle training; MIP, maximal inspiratory pressure; 6MWT, six-minute walk test; NR, not reported or not ascertainable; POD, postoperative day; PT, physical therapist; TAVR, transcatheter aortic valve replacement. TIME denotes the study-specific prehabilitation bundle described in the table.

## Table S6 Exercise frequency intensity duration and supervision

| **Study** | **Frequency** | **Intensity and progression** | **Time per session** | **Active programme duration** | **Supervision and setting** |
| --- | --- | --- | --- | --- | --- |
| Pressler 2016 | 2 sessions in week 1, then 3/week; resistance 2/week from week 2 | Cycling 40% to 70% peak VO2; resistance 30% to 50–60% 1-RM | Cycling 20–45 min; 1 × 10 to 3 × 15 repetitions | 8 weeks | Supervised at an exercise centre |
| Cargnin 2019 | Twice daily, 7 days/week | 30% MIP, adjusted weekly | 30 breaths/session | 4 weeks | Inpatient physiotherapy; home diary and weekly hospital review after discharge |
| Nilsson 2018 | 3 sessions/week | 40–60%, then 50–70%, then 60–80% baseline peak VO2 | 10-min warm-up + 20-min exercise + 5–10-min cool-down | 12 weeks | Supervised |
| Rogers 2018 | Once weekly for 6 sessions | Moderate-intensity intervals, progressed using Borg ratings | 60–90 min/session | 6 weekly sessions; extra sessions allowed | Exercise-team supervision; ECG and heart-rate monitoring |
| Sire 1987 | Daily during 4-week programme; exact days/week NR | Upper pulse limit 85–90% of initial test maximum heart rate | 3–4 hours/day, including an intermission | 4 weeks | Rehabilitation institute programme with socio-occupational team |
| Cao 2013 | NR | Low-intensity staged mobilization; prescribed target NR | NR | Inpatient phase; exact duration NR | Nurse-guided inpatient rehabilitation; session supervision NR |
| Li 2020 | Exercise once daily; repeated interviews | MOTOmed initial resistance 1 kg, individualized; other numerical targets NR | MOTOmed 30 min or other exercise 20 min; interviews about 20 min each | 7 preoperative days | Nursing/rehabilitation staff, BP/ECG/oxygen saturation monitoring |
| Lin 2004 | Breathing trainer twice daily; cough practice 3/day; home aerobic exercise 2–3/week | Home stretching 3–5 METs; aerobic exercise 5–7 METs; individualized strength work | Cough practice about 20 repetitions; stretching 3–5 min; aerobic 20–30 min | Preoperative week + inpatient phase + 3 months after discharge | Rehabilitation physician-led inpatient care; home programme after discharge |
| Sun 2021 | Once daily | Individualized by baseline 6MWT grade; numerical target NR | 30 min for grades 1–2; 20 min for grades 3–4 | 1 week | Nurse-guided with vital-sign and BP monitoring |
| Zou 2020 | 3/day initially; increased on POD 5–8 | Progressed according to tolerance; numerical target NR | NR | Inpatient rehabilitation; exact end date NR | Nurse-guided under ECG monitoring initially |
| Tao 2020 | Twice daily | RPE-guided stage progression; walking 30, 50, 100, 150 or 200 m | Initial 5–10 repetitions; simulated cycling 20 repetitions; minutes NR | Until day before discharge | Nurse-assisted, then medical-team-guided |
| Tamulevičiūtė-Prascienė 2021 | Additional resistance/balance 3/week; balance 2–3/week | Resistance <30% 1-RM/RPE ≤11, increasing to 30–60% 1-RM/RPE 12–13 | 5–10 to 8–15 repetitions, up to 3 sets; balance 10–15 min | 20-day inpatient CR; 12-week home advice/contact thereafter | Physiotherapist-supervised small groups of 3 |
| Weber 2021 | Preoperative IMT 4/day and walking daily; postoperative physiotherapy 2/day | Walking below subjective exhaustion; numerical IMT load NR | IMT 4 × 5 min/day; walking ≥30 min/day; postoperative 2 × 30 min/day | ≥2 weeks before TAVR through discharge | Ambulatory individualized prehabilitation with diary; supervised inpatient physiotherapy |
| Xue 2022 | 3 sessions/day | Limited by clinical status and tolerance | Approximately 30 min/session | POD 1 to discharge | Monitored and assisted by physical therapists |
| Xu 2022 | Once daily, 3–5 days/week | Initially 30% MIP, progressed to ≥60%; recovery phase <30% | 5-min warm-up + 10-min loaded IMT + 5-min recovery | During hospital stay | Supervised by therapists |
| Hu (ENERGY) 2023 | 3 sessions/week | Target HR at anaerobic threshold ±5 beats/min, reassessed monthly | 20 min progressed to 45 min at target intensity, plus warm-up/cool-down | 3 months | Month 1: 2 supervised + 1 home session/week; months 2–3: home-based with HR monitoring and telephone encouragement |
| Vitez 2023 | 2/week; 16–24 visits | Cycling 40% to 75% peak VO2; low/moderate resistance | 10-min warm-up + 20–40-min cycling + resistance/balance + 10-min cool-down | 8–12 weeks | Cardiovascular physiotherapist-supervised centre programme |
| Song 2024 | 5 days/week | Anaerobic-threshold HR or power; cycle power used for AF | 40 min/day: 5 warm-up, 30 exercise, 5 rest | 12 weeks | Monitored oxygen saturation, HR and BP during CR |
| Shi 2025 | 8 visits: twice/week in weeks 1–2, once/week in weeks 3–4, then weeks 6 and 8 | Individually progressed by physical therapist; numerical target NR | Exercise 40 min/visit; arm intervention group adds CBI 20 min/visit | 8 weeks | Physical therapist home visits plus independent exercise |
| Gao 2025 | Most mobility exercises twice daily; respiratory exercise 3/day preoperatively | Graded walking 50–200 m; physiological stopping rules rather than fixed HR training target | Early limb/cycling exercises 10–15 min twice daily; sputum therapy 20 min | Inpatient programme; follow-up support to 1 month after discharge | Multidisciplinary ERAS team with monitored ward/ICU mobilization |
| Liu 2025 | IMT twice/day; active-cycle breathing 3/day; postoperative mobilization twice/day | IMT starts 30% MIP, increments of 5% to RPE 11–13; activity RPE individualized | IMT 20 min/session; active-cycle breathing 10 min/session; mobility session length NR | Preoperative admission through discharge | Physical therapist supervision with pre-session safety assessment |
| Li 2023 | Breathing twice/day; passive movement 3/day; early walking twice/day; later aerobic/resistance session daily | Abdominal sandbag 1 kg; later reported 10–15% exercise intensity has no defined denominator | Breathing 40 min/session; passive movement 5 min; active activity 30 min; early walking 10 min; later exercise 40 min | 3 months of nursing intervention | Staff-guided nursing programme; monitoring reported |
| Gao 2024 | Limb exercise and simulated cycling twice/day; walking frequency NR | Progression by tolerance with HR/RR/BP monitoring; walking starts at 30 m | 5–10 repetitions for limb exercises; simulated cycling 20 min/session | Not clearly reported | Video guidance plus staff and family support |
| Zhang 2026 | Breathing/cough training 3–4/day; mobility frequency NR | Tolerance-based progression; prescribed physiological target NR | Breathing/coughing 3–5 min/session | Early inpatient period; endpoint NR | Nursing guidance with monitoring |
| Sibilitz 2016 | Exercise 3/week; consultations monthly × 5 | Borg-guided progressively increasing aerobic intensity; exact targets in protocol, not in this PDF | Exercise session length NR in supplied PDF | Exercise 12 weeks; five monthly psycho-educational consultations | First 1–3 sessions at hospital; thereafter supervised facility (69%) or home-based programme (31%) |
| Luo 2023 | IMT twice/day; programme reports 12 training sessions over 3 days; walking frequency not separately specified | IMT 30% MIP, +2 cmH2O if Borg <5; walking 60% heart-rate reserve derived from baseline 6MWT | IMT 20 min/session; walking 20 min/session; education 20 min after IMT | 3 preoperative days | Physiotherapist-led multidisciplinary team with cardiac-surgeon safety review |
| Yu 2025 | Twice daily | 30% MIP, +2 cmH2O if Borg <5; fixed 10% MIP | 20 min/session | 3 days planned; continued if surgery delayed | Senior physiotherapist supervision |
| Ahmad 2024 | Once daily | Work phase RPE 11–12; target HR resting HR +20–30 bpm | 15–20 min: warm-up 5 min, cycling 5–10 min, cool-down 5 min | POD 1 through hospital discharge | Direct physiotherapist supervision with vital-sign monitoring |
| Atef 2026 | 3 sessions/week | Aerobic and resistance RPE 13–15; resistance load adjusted by pain-free technique and fatigue, not 1-RM testing | Aerobic component 20→25→30 min; warm-up 15 min, cool-down 10 min; RT 1 × 12 repetitions of six exercises, 4:2:4 tempo | 4 weeks (12 sessions) | Physiotherapist-supervised with continuous ECG monitoring; inpatient then outpatient |

Abbreviations: AF, atrial fibrillation; BP, blood pressure; bpm, beats per minute; CBI, cognitive-behavioural intervention; CR, cardiac rehabilitation; ECG, electrocardiogram; ERAS, enhanced recovery after surgery; HR, heart rate; ICU, intensive care unit; IMT, inspiratory muscle training; METs, metabolic equivalents; MIP, maximal inspiratory pressure; 6MWT, six-minute walk test; NR, not reported or not ascertainable; POD, postoperative day; 1-RM, one-repetition maximum; RPE, rating of perceived exertion; RR, respiratory rate; RT, resistance training; TAVR, transcatheter aortic valve replacement; peak VO2, peak oxygen uptake; cmH2O, centimetres of water.

# Appendix 6 Rehabilitation stages and outcome assessment timing

## Table S7 Intervention initiation and rehabilitation stages

| **Study** | **Phase** | **Initiation** | **Preoperative component** | **Postoperative component and progression** | **Active programme duration** | **Reported assessments** |
| --- | --- | --- | --- | --- | --- | --- |
| Pressler 2016 | Postoperative | Mean 83 ± 34 days after TAVI | No study-specific preoperative training reported | Training began 42–132 days after TAVI, not during the immediate inpatient phase. | 8 weeks | End of 8-week training |
| Cargnin 2019 | Postoperative | Postoperative day 3 | No study-specific preoperative training reported | POD 3 through 4 weeks, including home training after discharge. | 4 weeks | After 4 weeks of IMT |
| Nilsson 2018 | Postoperative | 5–6 weeks after surgery | No study-specific preoperative training reported | Structured cycling began 5–6 weeks after AVR; advice continued after programme completion. | 12 weeks | End of 12-week training; 1 year thereafter |
| Rogers 2018 | Postoperative | 1 month after TAVI | No study-specific preoperative training reported | No formal CR before 1 month; study CR starts at 1 month. | 6 weekly sessions; extra sessions allowed | 3 and 6 months after randomization |
| Sire 1987 | Postoperative | 2 months after surgery | No study-specific preoperative training reported | Institutional training starts at 2 months; home exercises advised. | 4 weeks | 2, 6 and 12 months after surgery; end-of-training test in T only |
| Cao 2013 | Postoperative | During postoperative phase-I inpatient CR; exact day NR | No study-specific preoperative training reported | Phase-I programme; no study-specific preoperative exercise described. | Inpatient phase; exact duration NR | During admission; day before discharge |
| Li 2020 | Preoperative | 7 days before surgery | Seven-day exercise programme before surgery. | No differential postoperative exercise programme described; postoperative outcomes measured. | 7 preoperative days | LVEF: POD 7; 6MWD/ADL: 1 month after surgery |
| Lin 2004 | Perioperative | 1 week before surgery | Breathing/cough training, respiratory trainer and active joint movements from 1 week preoperatively; paused on surgery day. | POD 1 breathing/cough/passive legs; POD 2 passive limbs/turning; POD 3 active limbs/sitting; POD 4 onward standing/walking. Home training for 3 months. | Preoperative week + inpatient phase + 3 months after discharge | Discharge and 3 months after discharge |
| Sun 2021 | Preoperative | Before surgery; after admission | One-week exercise programme with repeat 6MWT afterwards. | No added postoperative training described. | 1 week | After nursing intervention; exact postoperative timing NR |
| Zou 2020 | Postoperative | POD 2–3 when stable | No study-specific preoperative training reported | Breathing/passive exercise POD 2–3; active limbs and out-of-bed walking POD 5–8. | Inpatient rehabilitation; exact end date NR | After nursing; exact day NR |
| Tao 2020 | Postoperative | Immediately postoperatively, while still ventilated | No study-specific preoperative training reported | Passive limbs during ventilation, active limbs/breathing after extubation, then cycling/walking. | Until day before discharge | Inpatient phase; before discharge |
| Tamulevičiūtė-Prascienė 2021 | Postoperative | Within 4 weeks; mean 14.5 days after procedure | No study-specific preoperative training reported | Additional resistance starts no earlier than CR day 3; subsequent 12-week follow-up contact is not 12 weeks of supervised training. | 20-day inpatient CR; 12-week home advice/contact thereafter | CR completion; 3 months after CR completion |
| Weber 2021 | Perioperative | At least 2 weeks before TAVR | Daily IMT and walking for at least 14 days; actual mean training period 30.2 ± 19.9 days. | Intensified mobilization from the evening of TAVR, twice daily until discharge. | ≥2 weeks before TAVR through discharge | Inpatient, 30 days and 90 days after TAVR |
| Xue 2022 | Postoperative | Postoperative day 1 | No study-specific preoperative training reported | POD 1 onward; study training stops at discharge, not at 6 months. | POD 1 to discharge | Discharge and 6 months after discharge |
| Xu 2022 | Postoperative | Post-TAVR inpatient phase; CR begins POD 2 | No study-specific preoperative training reported | Exercise CR begins POD 2. IMT given concurrently during admission; IMT-specific first day is not separately stated. | During hospital stay | Discharge; 1 and 3 months after discharge |
| Hu (ENERGY) 2023 | Postoperative | ≥1 month after TAVR; median 157 days (IQR 35–200.25) | No study-specific preoperative training reported | Delayed outpatient/home training after TAVR. | 3 months | 3 months after randomization |
| Vitez 2023 | Postoperative | 3–6 months after TAVI; mean 110 days reported | No study-specific preoperative training reported | Both groups receive exercise after TAVI; setting/supervision/content differ. | 8–12 weeks | After 8–12-week study period |
| Song 2024 | Postoperative | Within 1 week after baseline testing; exact interval from TAVR unclear | No study-specific preoperative training reported | Exercise starts within 1 week of baseline/randomization; paper states both recruitment within 24 h of TAVR and clinical stability >30 days. | 12 weeks | Within 1 week after 12-week CR |
| Shi 2025 | Postoperative | Within 14 days after discharge (Methods) | No study-specific preoperative training reported | Home rehabilitation after discharge. Methods specify ≤14 days; abstract says ≤7 days. | 8 weeks | 4 and 8 weeks after intervention start |
| Gao 2025 | Postoperative | Postoperative day 1 for the main between-arm contrast | Both arms receive preoperative management; §Methods explicitly states the same preoperative protocol. | Main contrast: earlier/intensified mobility and different pain/nutrition management after surgery. | Inpatient programme; follow-up support to 1 month after discharge | During admission; planned reviews at 1, 2 weeks and 1 month after discharge |
| Liu 2025 | Perioperative | Education on randomization day; exercise from the next day before surgery | Education at randomization; IMT and active-cycle breathing begin the next day before surgery. | IMT resumes on first cardiac-ward day; active-cycle breathing continues; mobilization begins POD 1 until discharge. | Preoperative admission through discharge | PPCs within 7 days; hospital outcomes; 3-month mortality |
| Li 2023 | Postoperative | Postoperatively, including the mechanical-ventilation phase; exact day NR | No study-specific preoperative training reported | Passive joint movement while ventilated, active ADL after extubation, then walking, stairs and more advanced exercise. | 3 months of nursing intervention | After 3-month intervention |
| Gao 2024 | Postoperative | Postoperative period; exact day NR | No study-specific preoperative training reported | Postoperative progression from passive movement to cycling and walking. | Not clearly reported | LVEF: day before discharge; 6MWD: after nursing, exact day NR; 3-month follow-up reported |
| Zhang 2026 | Postoperative | Within 12 hours after surgery | No study-specific preoperative training reported | Early staged postoperative exercise; no preoperative training described. | Early inpatient period; endpoint NR | After nursing intervention; exact day NR |
| Sibilitz 2016 | Postoperative | Exercise 1 month after surgery; consultations within the first month | No study-specific preoperative training reported | Study exercise begins at 1 month, not immediately after surgery. | Exercise 12 weeks; five monthly psycho-educational consultations | Physical outcomes 1 and 4 months; patient-reported outcomes through 6 months postrandomization |
| Luo 2023 | Preoperative | 3 days before the planned procedure | Only the TIME arm receives the 3-day additional prehabilitation bundle. | Both groups receive the same early mobilization/chest physiotherapy from POD 1. | 3 preoperative days | PPCs within 14 days; hospital outcomes; 3-month EQ-5D |
| Yu 2025 | Preoperative | 3 days before the planned procedure | Allocated training for 3 days; if operation delayed, allocated treatment continues until surgery. | All arms receive the same postoperative respiratory physiotherapy and mobilization. | 3 days planned; continued if surgery delayed | Day before surgery, discharge, and EQ-5D at 1 month after discharge |
| Ahmad 2024 | Postoperative | Postoperative day 1 in ICU | No study-specific preoperative training reported | Additional cycling begins POD 1 and ends at discharge; SF-12 follow-up does not extend exercise exposure. | POD 1 through hospital discharge | Discharge functional outcomes; SF-12 at 1 month after discharge |
| Atef 2026 | Postoperative | Within 48 hours after surgery if haemodynamically stable | No study-specific preoperative training reported | Begins within 48 h and continues after hospital discharge to 4 weeks. | 4 weeks (12 sessions) | After 4-week intervention; inpatient stay outcomes |

Abbreviations: T, intervention group; ADL, activities of daily living; AVR, aortic valve replacement; CR, cardiac rehabilitation; EQ-5D, EuroQol five-dimension questionnaire; ICU, intensive care unit; IMT, inspiratory muscle training; IQR, interquartile range; LVEF, left ventricular ejection fraction; 6MWD, six-minute walk distance; 6MWT, six-minute walk test; NR, not reported or not ascertainable; POD, postoperative day; PPC, postoperative pulmonary complication; SF-12, 12-item Short Form Health Survey; TAVR/TAVI, transcatheter aortic valve replacement/implantation. TIME denotes the study-specific prehabilitation bundle described in Table S5.

Functional outcome windows are anchored to the valve procedure: ≤30 days, 31–90 days and >90 days. A month is treated as 30 days for window arithmetic. Unknown anchors, ranges crossing a cutoff, and discharge without a postoperative day are classified as unclear.

## Table S8 Selected functional outcomes and assessment windows

| **Outcome** | **Study** | **Selected assessment** | **Postprocedure window** | **Timing note** |
| --- | --- | --- | --- | --- |
| 6MWD | Pressler 2016 | End of 8-week training | >90 days | Training started 42–132 days after TAVI. |
| 6MWD | Cargnin 2019 | End of 4-week training | Unclear | Training starts on POD 3; assessment is near the 1-month cutoff, with no exact day reported. |
| 6MWD | Rogers 2018 | 6 months after randomisation | >90 days | Randomisation followed TAVI. |
| 6MWD | Li 2020 | 1 month after surgery | ≤30 days | Procedure-anchored timing available. |
| 6MWD | Sun 2021 | After nursing; date NR | Unclear | Postoperative interval not established. |
| 6MWD | Tamulevičiūtė-Prascienė 2021 | End of approximately 20-day CR | Unclear | CR starts within 4 postoperative weeks; the interval crosses the 1-month cutoff. |
| 6MWD | Xue 2022 | Hospital discharge | Unclear | Discharge day NR; not assumed to be within 1 month. |
| 6MWD | Xu 2022 | 3 months after discharge | >90 days | Window is anchored to TAVR, not discharge. |
| 6MWD | Hu (ENERGY) 2023 | 3 months after randomisation | >90 days | Randomisation was ≥1 month after TAVR. |
| 6MWD | Vitez 2023 | End of 8–12-week programme | >90 days | Training started 3–6 months after TAVI. |
| 6MWD | Song 2024 | Within 1 week after 12-week CR | Unclear | Interval between TAVR and programme start unclear. |
| 6MWD | Gao 2025 | Postoperative day 5 | ≤30 days | Procedure-anchored timing available. |
| 6MWD | Li 2023 | After 3 months of nursing | Unclear | Exact start date and postoperative interval NR. |
| 6MWD | Gao 2024 | After nursing; date NR | Unclear | The day-before-discharge timing is explicit for LVEF only. |
| 6MWD | Zhang 2026 | After nursing; date NR | Unclear | Exact date not documented for this selected result. |
| 6MWD | Sibilitz 2016 | 4 months after randomisation | >90 days | Procedure-anchored timing available. |
| 6MWD | Yu 2025 | Hospital discharge | Unclear | Discharge day NR; not assumed to be within 1 month. |
| 6MWD | Ahmad 2024 | Hospital discharge | Unclear | Discharge day NR; not assumed to be within 1 month. |
| 6MWD | Atef 2026 | End of 4-week training | ≤30 days | Training starts within 48 h; approximately POD 28–30. |
| Peak VO₂ | Pressler 2016 | End of 8-week training | >90 days | Training started 42–132 days after TAVI. |
| Peak VO₂ | Nilsson 2018 | End of 12-week training | >90 days | Training started 5–6 weeks after surgery. |
| Peak VO₂ | Tamulevičiūtė-Prascienė 2021 | 3 months after CR completion | >90 days | CR completion was after the valve procedure. |
| Peak VO₂ | Xu 2022 | 3 months after discharge | >90 days | Window is anchored to TAVR, not discharge. |
| Peak VO₂ | Hu (ENERGY) 2023 | 3 months after randomisation | >90 days | Randomisation was ≥1 month after TAVR. |
| Peak VO₂ | Vitez 2023 | End of 8–12-week programme | >90 days | Training started 3–6 months after TAVI. |
| Peak VO₂ | Song 2024 | Within 1 week after 12-week CR | Unclear | Interval between TAVR and programme start unclear. |
| Peak VO₂ | Sibilitz 2016 | 4 months after randomisation | >90 days | Procedure-anchored timing available. |
| LVEF | Pressler 2016 | End of 8-week training | >90 days | Training started 42–132 days after TAVI. |
| LVEF | Cargnin 2019 | Selected LVEF assessment date not established | Unclear | Do not infer an endpoint date from trial duration or a clinical-characteristics table. Excluded from timing-restricted analyses. |
| LVEF | Li 2020 | Postoperative day 7 | ≤30 days | Procedure-anchored timing available. |
| LVEF | Sun 2021 | After nursing; date NR | Unclear | Postoperative interval not established. |
| LVEF | Zou 2020 | After nursing; date NR | Unclear | Exact date not documented for this selected result. |
| LVEF | Tamulevičiūtė-Prascienė 2021 | Selected LVEF assessment date not established | Unclear | Exact postoperative day of the selected LVEF assessment was not reported and was not inferred from trial duration |
| LVEF | Weber 2021 | Selected LVEF assessment date not established | Unclear | Exact postoperative day of the selected LVEF assessment was not reported and was not inferred from trial duration |
| LVEF | Xue 2022 | Selected LVEF assessment date not established | Unclear | Exact postoperative day of the selected LVEF assessment was not reported and was not inferred from trial duration |
| LVEF | Xu 2022 | 3 months after discharge | >90 days | Window is anchored to TAVR, not discharge. |
| LVEF | Hu (ENERGY) 2023 | 3 months after randomisation | >90 days | Randomisation was ≥1 month after TAVR. |
| LVEF | Song 2024 | Within 1 week after 12-week CR | Unclear | Interval between TAVR and programme start unclear. |
| LVEF | Li 2023 | After 3 months of nursing | Unclear | Exact start date and postoperative interval NR. |
| LVEF | Gao 2024 | Day before hospital discharge | Unclear | Discharge day NR; not assumed to be within 1 month. |
| LVEF | Zhang 2026 | After nursing; date NR | Unclear | Exact date not documented for this selected result. |

Abbreviations: CR, cardiac rehabilitation; LVEF, left ventricular ejection fraction; 6MWD, six-minute walk distance; NR, not reported or not ascertainable; POD, postoperative day; peak VO₂, peak oxygen uptake; TAVR/TAVI, transcatheter aortic valve replacement/implantation.

# Appendix 7 Safety outcome definitions

## Table S9 Adverse event definitions and observation periods

| **Study** | **Reported definition or components** | **Observation period** | **Clinical construct** |
| --- | --- | --- | --- |
| Pressler 2016 | Clinical events interrupting training for >1 week or ending participation; training-related events occurred during or within 12 h after exercise. | 8 weeks | Training-related AEs; separate SAEs |
| Cargnin 2019 | Exercise intolerance (dizziness, pallor, dyspnoea, hypotension), sternal/wound problems and arrhythmias. | 4 weeks | Training/wound safety |
| Rogers 2018 | No rehabilitation-associated AEs reported; definition NR. | AE window NR; trial follow-up 6 months | Training-associated safety |
| Sire 1987 | Abdominal muscle haematoma during training; non-fatal thromboembolic episodes in controls. Common AE definition NR. | Up to 12 months after surgery | Separate clinical events |
| Cao 2013 | Postoperative complications; components and criteria NR. | Postoperative hospital stay | General postoperative morbidity |
| Li 2020 | Postoperative complications; components and criteria NR. | Perioperative admission; exact endpoint NR | General postoperative morbidity |
| Lin 2004 | Pulmonary complications (see Table S10). | Postoperative hospital stay | Pulmonary morbidity |
| Zou 2020 | Postoperative complications; components and criteria NR. | Nursing period; exact endpoint NR | General postoperative morbidity |
| Tao 2020 | Nausea/vomiting, choking/coughing, abdominal distension and hypoglycaemia. | After surgery to the day before discharge | Postoperative morbidity |
| Xu 2022 | Cerebrovascular events, permanent pacemaker implantation, pulmonary infection and urinary tract infection; no treatment-related AEs reported separately. | 3 months after valve replacement | Postoperative morbidity; separate safety statement |
| Vitez 2023 | Training-related adverse events; a common prospective definition and complete surveillance of all assigned participants are not established. | 8–12-week programme | Training-associated safety; incomplete ascertainment |
| Song 2024 | Severe arrhythmia, acute coronary syndrome, syncope or sudden death during exercise testing or rehabilitation. | 12 weeks | Serious testing/training-associated events |
| Shi 2025 | AE: any unfavourable sign, symptom or disease, irrespective of causality. SAE: death, life-threatening event, hospitalisation/prolongation or persistent/significant disability. | 8 weeks from the first visit | All-cause AEs; SAEs separately |
| Gao 2025 | AE composite from hospital records; components NR. Pneumonia reported separately (Table S10). | Hospital records; exact window NR | Undefined clinical AE composite |
| Li 2023 | Muscle atrophy, pressure ulcers and lower-limb deep-vein thrombosis. | 3 months | Postoperative morbidity |
| Gao 2024 | Arrhythmia, heart failure, angina and revascularisation. | Follow-up 3 months; exact AE window NR | General cardiovascular events |
| Sibilitz 2016 | Self-reported non-serious events: recurrent pericardial effusion, palpitations, dyspnoea, thromboembolic symptoms, chest pain and musculoskeletal injuries. Intervention-group SAEs were judged unrelated to rehabilitation. | 6 months | Non-serious AEs; SAEs separately |
| Yu 2025 | AEs during preoperative testing or training; a common prospective definition for the selected binary endpoint is not fully documented. | Preoperative testing/training | Testing/training safety |
| Atef 2026 | Exercise-related AEs: non-life-threatening events reducing exercise tolerance (fatigue, muscle soreness, dyspnoea). SAEs: events requiring medical intervention, causing disability or death. | 4 weeks (12 sessions) | Exercise-related AEs; SAEs separately |

AE, adverse event; SAE, serious adverse event. NR indicates that a definition, criterion or exact observation period was not reported or could not be established. Absence of reported training-related events does not establish complete surveillance for all clinical events.

## Table S10 Pulmonary complication definitions and observation periods

| **Study** | **Reported definition or components** | **Observation period** | **Criteria or reporting limitation** |
| --- | --- | --- | --- |
| Lin 2004 | Atelectasis, pleural effusion and pneumonia; diagnostic criteria NR. | Postoperative hospital stay | PPC composite; pneumonia separately |
| Weber 2021 | Kroenke PPC score >2. Pneumonia: radiological diagnosis, with or without bacteriological confirmation. | Postprocedural hospital stay | PPC composite; pneumonia separately |
| Shi 2025 | Pulmonary events recorded within the general AE framework; an outcome-specific diagnostic or adjudication protocol is not established. | 8-week intervention | Pulmonary-event definition and surveillance incompletely specified |
| Gao 2025 | Physician-diagnosed pneumonia based on chest radiography/CT and clinical signs. | Hospital records; exact endpoint NR | Pneumonia only |
| Liu 2025 | Atelectasis, pneumothorax, pleural effusion, respiratory failure and pneumonia. | First 7 postoperative days | PPC composite; pneumonia separately |
| Luo 2023 | Hulzebos/Kroenke criteria: ≥2 grade 2 complications or ≥1 grade 3 or 4 complication. | Within 14 days after surgery | Clinically significant PPC composite |
| Yu 2025 | Kroenke criteria: ≥2 grade 2 complications or ≥1 grade 3 or 4 complication. | Postoperative hospital stay | Clinically significant PPC composite |

Abbreviations: AE, adverse event; CT, computed tomography; NR, not reported or not ascertainable; PPC, postoperative pulmonary complication.

# Appendix 8 Outcome specific risk of bias

RoB 2 assessments target the effect of assignment to intervention for each selected outcome and assessment time. Of 83 selected results across 29 trials, 17 were rated high risk and 66 as some concerns; none was at overall low risk. High-risk exclusions in Table S15 are outcome-specific. For Gao 2025, the report does not clearly establish whether allocation occurred at the patient or ward/floor level; the retained judgments and a separate exclusion analysis reflect this design uncertainty.

## Table S11 RoB 2 judgments for the selected results

| **Outcome** | **Study** | **Selected result or time** | **D1** | **D2** | **D3** | **D4** | **D5** | **Overall** | **Supporting reason** |
| --- | --- | --- | --- | --- | --- | --- | --- | --- | --- |
| 6MWD | Pressler 2016 | End of 8-week training | SC | L | SC | L | SC | SC | Concealment unclear; 27/30 assessed after health-related withdrawals. |
| 6MWD | Cargnin 2019 | End of 4-week training | SC | L | L | L | SC | SC | Concealment unclear; sham control, blinded assessment and complete follow-up. |
| 6MWD | Rogers 2018 | 6 months after randomisation | L | L | H | L | SC | H | Only 14/27 could perform the test; frailty prevented testing. |
| 6MWD | Li 2020 | 1 month after surgery | SC | SC | L | SC | SC | SC | Concealment, intervention deviations and assessor masking unclear. |
| 6MWD | Sun 2021 | After nursing; date NR | SC | SC | L | SC | SC | SC | Concealment, deviations and assessor masking unclear; timing unspecified. |
| 6MWD | Tamulevičiūtė-Prascienė 2021 | End of approximately 20-day CR | SC | L | L | L | SC | SC | Concealment unclear; 113/116 completed short-term assessment. |
| 6MWD | Xue 2022 | Hospital discharge | L | L | L | L | SC | SC | All 87 assessed at discharge; blinded assessors. |
| 6MWD | Xu 2022 | 3 months after discharge | SC | L | SC | L | SC | SC | Concealment unclear; 62/96 assessed, with unequal follow-up between arms. |
| 6MWD | Hu (ENERGY) 2023 | 3 months after randomisation | SC | L | L | L | SC | SC | Sequence/concealment details incomplete; 64/66 assessed; blinded assessors. |
| 6MWD | Vitez 2023 | End of 8–12-week programme | L | L | H | SC | SC | H | 23/39 completed; health-related withdrawals; assessor masking unclear. |
| 6MWD | Song 2024 | Within 1 week after 12-week CR | SC | SC | L | SC | SC | SC | Randomisation safeguards and deviations unclear; unblinded assessment. |
| 6MWD | Gao 2025 | Postoperative day 5 | SC | SC | SC | SC | SC | SC | Patient randomisation reported; unit unclear; 6 withdrawals excluded; unblinded ward assessment. |
| 6MWD | Li 2023 | After 3 months of nursing | SC | SC | L | SC | SC | SC | Concealment, deviations and assessor masking unclear. |
| 6MWD | Gao 2024 | After nursing; date NR | SC | SC | SC | SC | SC | SC | Concealment/deviations unclear; complete-record selection; masking unclear. |
| 6MWD | Zhang 2026 | After nursing; date NR | SC | SC | SC | SC | SC | SC | Withdrawal exclusions unclear; concealment, deviations and masking unclear. |
| 6MWD | Sibilitz 2016 | 6MWD at four months after randomisation | L | L | H | L | SC | H | Health-related losses can depend on walking capacity; no 6MWD-specific analysis demonstrates robustness to nonrandom missingness. |
| 6MWD | Yu 2025 | Hospital discharge | L | L | L | L | SC | SC | Concealed allocation; sham comparison; blinded assessment; complete data. |
| 6MWD | Ahmad 2024 | Hospital discharge | L | SC | H | SC | SC | H | Nonrecipients excluded; early discharge caused missing tests; unblinded. |
| 6MWD | Atef 2026 | End of 4-week training | L | L | L | L | SC | SC | Concealed allocation; blinded assessor; all 40 randomised patients completed. |
| Peak VO₂ | Pressler 2016 | End of 8-week training | SC | L | SC | L | SC | SC | Concealment unclear; 27/30 assessed after health-related withdrawals. |
| Peak VO₂ | Nilsson 2018 | End of 12-week training | SC | L | L | SC | SC | SC | Randomisation safeguards and assessor masking unclear; all 12 assessed. |
| Peak VO₂ | Tamulevičiūtė-Prascienė 2021 | 3 months after CR completion | SC | L | H | L | SC | H | Concealment unclear; only 76/116 returned; illness/deaths among losses. |
| Peak VO₂ | Xu 2022 | 3 months after discharge | SC | L | SC | L | SC | SC | Concealment unclear; substantial, unequal follow-up loss; blinded testing. |
| Peak VO₂ | Hu (ENERGY) 2023 | 3 months after randomisation | SC | L | L | L | SC | SC | Sequence/concealment details incomplete; 64/66 assessed; blinded testing. |
| Peak VO₂ | Vitez 2023 | End of 8–12-week programme | L | L | H | SC | SC | H | 23/39 completed; health-related withdrawals; assessor masking unclear. |
| Peak VO₂ | Song 2024 | Within 1 week after 12-week CR | SC | SC | L | SC | SC | SC | Randomisation safeguards and deviations unclear; unblinded testing. |
| Peak VO₂ | Sibilitz 2016 | 4 months after randomisation | L | L | H | L | L | H | 63/72 vs 52/75 tested; health-related missingness; imputation changed inference. |
| LVEF | Pressler 2016 | End of 8-week training | SC | L | SC | SC | SC | SC | Concealment/echo masking unclear; health-related withdrawals. |
| LVEF | Cargnin 2019 | Selected LVEF result; follow-up date unconfirmed | SC | L | SC | SC | SC | SC | Selected LVEF assessment time and result-specific completeness are unconfirmed; concealment and echo details are insufficient. |
| LVEF | Li 2020 | Postoperative day 7 | SC | SC | L | SC | SC | SC | Concealment, deviations and echo assessor masking unclear. |
| LVEF | Sun 2021 | After nursing; date NR | SC | SC | L | SC | SC | SC | Concealment, deviations and echo masking unclear; timing unspecified. |
| LVEF | Zou 2020 | After nursing; date NR | SC | SC | L | SC | SC | SC | Randomisation/deviations unclear; claimed blinding insufficiently described. |
| LVEF | Tamulevičiūtė-Prascienė 2021 | Selected LVEF result; follow-up date unconfirmed | SC | L | SC | L | SC | SC | Selected LVEF visit is unconfirmed; endpoint-specific missingness cannot be assigned from the short- or medium-term flow. |
| LVEF | Weber 2021 | Selected LVEF result; follow-up date unconfirmed | SC | L | SC | SC | SC | SC | The selected LVEF assessment date, completeness and assessor masking are not sufficiently documented. |
| LVEF | Xue 2022 | Selected postoperative LVEF result; date unconfirmed | L | L | SC | L | SC | SC | LVEF is labelled postoperative without an assessment day; result-specific follow-up completeness is unclear. |
| LVEF | Xu 2022 | 3 months after discharge | SC | L | SC | L | SC | SC | Concealment unclear; substantial, unequal follow-up loss; blinded assessment. |
| LVEF | Hu (ENERGY) 2023 | 3 months after randomisation | SC | L | L | L | SC | SC | Sequence/concealment details incomplete; 64/66 assessed; blinded assessment. |
| LVEF | Song 2024 | Within 1 week after 12-week CR | SC | SC | L | SC | SC | SC | Randomisation safeguards and deviations unclear; unblinded echocardiography. |
| LVEF | Li 2023 | After 3 months of nursing | SC | SC | L | SC | SC | SC | Concealment, deviations and echo assessor masking unclear. |
| LVEF | Gao 2024 | Day before hospital discharge | SC | SC | SC | SC | SC | SC | Complete-record selection; allocation/deviations, measurement and masking unclear. |
| LVEF | Zhang 2026 | After nursing; date NR | SC | SC | SC | SC | SC | SC | Withdrawal exclusions unclear; concealment, deviations and masking unclear. |
| Hospital stay | Cargnin 2019 | Index postoperative hospital stay | SC | L | L | L | SC | SC | Concealment unclear; all randomised patients followed. |
| Hospital stay | Cao 2013 | Index postoperative hospital stay | SC | SC | L | L | SC | SC | Sequence/concealment and intervention deviations insufficiently reported. |
| Hospital stay | Li 2020 | Index postoperative hospital stay | SC | SC | L | L | SC | SC | Concealment and intervention deviations insufficiently reported. |
| Hospital stay | Lin 2004 | Index postoperative hospital stay | SC | SC | H | L | SC | H | Deviations/concealment unclear; health-related losses excluded from analysis. |
| Hospital stay | Sun 2021 | Index postoperative hospital stay | SC | SC | L | L | SC | SC | Sequence/concealment and intervention deviations insufficiently reported. |
| Hospital stay | Zou 2020 | Index postoperative hospital stay | SC | SC | L | L | SC | SC | Randomisation safeguards and intervention deviations insufficiently reported. |
| Hospital stay | Tao 2020 | Index postoperative hospital stay | SC | SC | L | L | SC | SC | Concealment and intervention deviations insufficiently reported. |
| Hospital stay | Weber 2021 | Index postoperative hospital stay | SC | L | L | L | SC | SC | Concealment unclear; complete inpatient data. |
| Hospital stay | Xue 2022 | Index postoperative hospital stay | L | L | L | L | SC | SC | All 87 assessed at discharge; later losses do not affect inpatient stay. |
| Hospital stay | Xu 2022 | Index postoperative hospital stay | SC | L | L | L | SC | SC | Concealment unclear; complete discharge data despite later follow-up loss. |
| Hospital stay | Gao 2025 | Index postoperative hospital stay | SC | SC | SC | L | SC | SC | Allocation unit/deviations unclear; 6 withdrawals excluded without reasons. |
| Hospital stay | Liu 2025 | Index postoperative hospital stay | L | H | L | L | SC | H | 160/978 excluded after randomisation; analysis restricted to operated patients. |
| Hospital stay | Luo 2023 | Index postoperative hospital stay | L | L | SC | L | SC | SC | Health-related withdrawals; missing outcomes imputed; nonrandom loss unresolved. |
| Hospital stay | Yu 2025 | Index postoperative hospital stay | L | L | L | L | SC | SC | Concealed allocation; complete data; blinded clinical assessment. |
| Hospital stay | Ahmad 2024 | Index postoperative hospital stay | L | SC | H | L | SC | H | Nonrecipients excluded; early discharge itself caused exclusion from analysis. |
| Hospital stay | Atef 2026 | Index postoperative hospital stay | L | L | L | L | SC | SC | Concealed allocation; all 40 randomised patients completed. |
| Adverse events | Pressler 2016 | 8 weeks | SC | L | SC | SC | SC | SC | Concealment unclear; surveillance completeness and assessor masking unclear. |
| Adverse events | Cargnin 2019 | 4 weeks | SC | L | L | L | SC | SC | Concealment unclear; both groups monitored; blinded, complete follow-up. |
| Adverse events | Rogers 2018 | Reported AEs over trial follow-up; exact event window not specified | L | L | L | SC | SC | SC | Follow-up retained, but equivalent AE surveillance and definitions in both arms are unclear. |
| Adverse events | Sire 1987 | Reported AEs through up to 12 months after surgery | SC | SC | H | SC | SC | H | Health-related exclusions, including angina, threaten complete assignment-based ascertainment of adverse events. |
| Adverse events | Cao 2013 | Postoperative hospital stay | SC | SC | L | SC | SC | SC | Allocation/deviations unclear; event definition and assessor masking unclear. |
| Adverse events | Li 2020 | Perioperative admission; exact endpoint NR | SC | SC | L | SC | SC | SC | Concealment/deviations unclear; event definition and assessor masking unclear. |
| Adverse events | Lin 2004 | Postoperative complications during the index hospital stay | SC | SC | H | SC | SC | H | Health-related withdrawals excluded; ascertainment and masking for postoperative complications are unclear. |
| Adverse events | Zou 2020 | Nursing period; exact endpoint NR | SC | SC | L | SC | SC | SC | Randomisation/deviations unclear; definition and effective masking unclear. |
| Adverse events | Tao 2020 | After surgery to the day before discharge | SC | SC | L | SC | SC | SC | Concealment/deviations unclear; subjective symptoms and unclear masking. |
| Adverse events | Xu 2022 | 3 months after valve replacement | SC | L | SC | L | SC | SC | Concealment unclear; substantial follow-up loss; blinded assessment reported. |
| Adverse events | Vitez 2023 | Reported training AEs during the 8-12-week programme | L | L | H | SC | SC | H | Safety reporting is centred on completers despite health-related withdrawals; surveillance of all randomised participants is unclear. |
| Adverse events | Song 2024 | 12 weeks | SC | SC | L | L | SC | SC | Randomisation/deviations unclear; severe events monitored during testing/training. |
| Adverse events | Shi 2025 | 8 weeks from the first visit | SC | H | SC | SC | SC | H | Three post-randomisation health/discharge exclusions; incomplete, partly unblinded surveillance. |
| Adverse events | Gao 2025 | Hospital records; exact window NR | SC | SC | SC | SC | SC | SC | Unit, deviations and withdrawal reasons unclear; event definition unclear. |
| Adverse events | Li 2023 | 3 months | SC | SC | L | SC | SC | SC | Concealment/deviations unclear; diagnostic criteria and assessor masking unclear. |
| Adverse events | Gao 2024 | Follow-up 3 months; exact AE window NR | SC | SC | SC | SC | SC | SC | Complete-record selection; allocation/deviations and assessor masking unclear. |
| Adverse events | Sibilitz 2016 | 6 months | L | L | SC | SC | SC | SC | Questionnaire completion unclear; unblinded patient reporting. |
| Adverse events | Yu 2025 | Preoperative testing/training AEs | L | L | L | SC | SC | SC | Complete assignment-based follow-up, but training AEs were recorded by unblinded therapists and reporting details are limited. |
| Adverse events | Atef 2026 | 4 weeks (12 sessions) | L | L | L | SC | SC | SC | Complete follow-up; subjective symptoms reported by unblinded participants. |
| Pulmonary complications | Lin 2004 | Postoperative hospital stay | SC | SC | H | SC | SC | H | Health-related losses excluded; concealment/deviations and diagnostic masking unclear. |
| Pulmonary complications | Weber 2021 | Postprocedural hospital stay | SC | L | L | SC | SC | SC | Concealment unclear; diagnostic criteria reported but assessor masking unclear. |
| Pulmonary complications | Shi 2025 | Pulmonary events during the eight-week intervention | SC | H | SC | SC | SC | H | Post-randomisation clinical exclusions threaten the assignment effect; pulmonary-event ascertainment is incompletely specified. |
| Pulmonary complications | Gao 2025 | Hospital records; exact endpoint NR | SC | SC | SC | SC | SC | SC | Unit, deviations and withdrawal reasons unclear; diagnosis unblinded. |
| Pulmonary complications | Liu 2025 | First 7 postoperative days | L | H | L | L | SC | H | 160/978 excluded after randomisation; blinded adjudication in operated patients. |
| Pulmonary complications | Luo 2023 | Within 14 days after surgery | L | L | SC | L | SC | SC | Health-related withdrawals; imputed outcomes; blinded assessors. |
| Pulmonary complications | Yu 2025 | Postoperative hospital stay | L | L | L | L | SC | SC | Concealed allocation; complete data; blinded assessment. |

Abbreviations: RoB 2, revised Cochrane risk-of-bias tool for randomized trials; D1, randomisation; D2, deviations from intended interventions; D3, missing outcome data; D4, outcome measurement; D5, selection of the reported result; L, low risk; SC, some concerns; H, high risk; AE, adverse event; CR, cardiac rehabilitation; LVEF, left ventricular ejection fraction; 6MWD, six-minute walk distance; NR, not reported or not ascertainable; peak VO₂, peak oxygen uptake.

# Appendix 9 Study grouping for subgroup analyses

Duration denotes the structured training course, excluding subsequent advice, telephone contact, and outcome follow-up. Studies with an unclear course length are omitted only from duration comparisons. Luo 2023 contains both surgical and TAVR participants and is omitted only from procedure-specific comparisons when its effect cannot be separated by procedure; it remains in the principal analyses and other applicable subgroup comparisons.

## Table S12 Grouping codes for the 29 included studies

| **Study** | **Procedure** | **Modality** | **Structured course** | **Duration group** | **Phase** | **Programme initiation** |
| --- | --- | --- | --- | --- | --- | --- |
| Pressler 2016 | TAVR | COMB | 8 weeks | ≤8 weeks | Postoperative | 42–132 days after TAVI |
| Cargnin 2019 | Surgery | RESP | 4 weeks | ≤8 weeks | Postoperative | Postoperative day 3 |
| Nilsson 2018 | Surgery | AER | 12 weeks | >8 weeks | Postoperative | 5–6 weeks after surgery |
| Rogers 2018 | TAVR | COMB | 6 weekly sessions | ≤8 weeks | Postoperative | 1 month after TAVI |
| Sire 1987 | Surgery | MULTI | 4 weeks | ≤8 weeks | Postoperative | 2 months after surgery |
| Cao 2013 | Surgery | MULTI | Inpatient programme; length NR | Unclear | Postoperative | Postoperative inpatient phase |
| Li 2020 | Surgery | MULTI | 7 days | ≤8 weeks | Preoperative | 7 days before surgery |
| Lin 2004 | Surgery | MULTI | Preoperative week through 3 months after discharge | >8 weeks | Perioperative | 1 week before surgery |
| Sun 2021 | Surgery | COMB | 1 week | ≤8 weeks | Preoperative | Before surgery |
| Zou 2020 | Surgery | MULTI | Inpatient programme; length NR | Unclear | Postoperative | Postoperative days 2–3 |
| Tao 2020 | Surgery | MULTI | Until the day before discharge | Unclear | Postoperative | Immediately after surgery |
| Tamulevičiūtė-Prascienė 2021 | Surgery | COMB | 20 days of structured CR | ≤8 weeks | Postoperative | Within 4 weeks after procedure |
| Weber 2021 | TAVR | MULTI | ≥2 weeks before TAVR through discharge | Unclear | Perioperative | ≥2 weeks before TAVR |
| Xue 2022 | Surgery | MULTI | Postoperative day 1 to discharge | Unclear | Postoperative | Postoperative day 1 |
| Xu 2022 | TAVR | RESP | During hospital stay | Unclear | Postoperative | Post-TAVR inpatient phase |
| Hu (ENERGY) 2023 | TAVR | AER | 3 months | >8 weeks | Postoperative | ≥1 month after TAVR |
| Vitez 2023 | TAVR | COMB | 8–12 weeks | Unclear | Postoperative | 3–6 months after TAVI |
| Song 2024 | TAVR | AER | 12 weeks | >8 weeks | Postoperative | Exact interval after TAVR unclear |
| Shi 2025 | TAVR | MULTI | 8 weeks | ≤8 weeks | Postoperative | Within 14 days after discharge |
| Gao 2025 | Surgery | MULTI | Inpatient programme; length NR | Unclear | Postoperative | Postoperative day 1 |
| Liu 2025 | Surgery | MULTI | Preoperative admission to discharge | Unclear | Perioperative | Before surgery; exact interval NR |
| Li 2023 | Surgery | MULTI | 3 months | >8 weeks | Postoperative | Postoperative ventilation phase |
| Gao 2024 | Surgery | MULTI | NR | Unclear | Postoperative | After surgery; exact day NR |
| Zhang 2026 | Surgery | MULTI | Early inpatient period; endpoint NR | Unclear | Postoperative | Within 12 hours after surgery |
| Sibilitz 2016 | Surgery | MULTI | 12 weeks of exercise | >8 weeks | Postoperative | Exercise starts 1 month after surgery |
| Luo 2023 | Mixed | MULTI | 3 days | ≤8 weeks | Preoperative | 3 days before planned valve procedure |
| Yu 2025 | Surgery | RESP | 3 days planned | ≤8 weeks | Preoperative | 3 days before planned surgery |
| Ahmad 2024 | Surgery | AER | Postoperative day 1 to discharge | Unclear | Postoperative | Postoperative day 1 |
| Atef 2026 | Surgery | COMB | 4 weeks | ≤8 weeks | Postoperative | Within 48 hours after surgery |

Abbreviations: AER, aerobic training; COMB, combined exercise; RESP, respiratory training or inspiratory muscle training; MULTI, early mobilisation or multimodal rehabilitation; CR, cardiac rehabilitation; NR, not reported or not ascertainable; TAVR/TAVI, transcatheter aortic valve replacement/implantation.

# Appendix 10 Main analysis estimates and prediction intervals

Principal analyses use DerSimonian–Laird random-effects models with Wald 95% confidence intervals. Continuous outcomes use inverse-variance MDs or SMDs (Hedges’ g); binary outcomes use the RevMan Mantel–Haenszel random-effects RR option. Approximate prediction intervals were calculated separately as the pooled effect ± t(0.975, k−1) × √[τ² + SE(M)²], where k is the number of estimable trials. RR intervals were calculated on the log scale and back-transformed. These intervals are approximate, especially with few studies.

## Table S13 Main pooled results

| **Outcome** | **Trials entered / estimable** | **Participants** | **Effect estimate 95% CI** | **P value** | **I² %** | **τ²** | **95% prediction interval** |
| --- | --- | --- | --- | --- | --- | --- | --- |
| 6MWD (MD, m) | 19 / 19 | 1574 | 40.95 (27.10 to 54.81) | <0.001 | 90.1 | 690.835 | -16.23 to 98.13 |
| Peak VO₂ (SMD) | 8 / 8 | 518 | 0.32 (0.07 to 0.57) | 0.014 | 46.2 | 0.058 | -0.33 to 0.97 |
| LVEF (MD, pp) | 14 / 14 | 1197 | 4.41 (2.26 to 6.56) | <0.001 | 89.5 | 13.564 | -3.89 to 12.71 |
| Hospital stay (MD, days) | 16 / 16 | 2327 | -1.77 (-2.41 to -1.13) | <0.001 | 90.8 | 1.234 | -4.24 to 0.69 |
| Adverse events (RR) | 19 / 14 | 1438 | 0.53 (0.33 to 0.85) | 0.009 | 44.2 | 0.319 | 0.14 to 1.99 |
| Pulmonary complications (RR) | 7 / 7 | 1554 | 0.62 (0.50 to 0.77) | <0.001 | 17.9 | 0.015 | 0.42 to 0.93 |

MD, mean difference; SMD, standardized mean difference; RR, risk ratio; pp, percentage points. Double-zero trials remain in the dataset but do not contribute to RR estimates or subgroup interaction tests. Participant counts describe the entered outcome dataset and can differ from the estimable subset. For adverse events, 19 trials contain 1438 participants; the RR is estimable in 14 trials containing 1276 participants. CI, confidence interval; LVEF, left ventricular ejection fraction; 6MWD, six-minute walk distance; peak VO₂, peak oxygen uptake. I², percentage of variability attributable to heterogeneity; τ², between-study variance; M, pooled effect; SE(M), standard error of the pooled effect.

# Appendix 11 Subgroup and sensitivity analyses

Subgroup analyses use separate heterogeneity estimates for each subgroup and formal χ² tests of subgroup differences. All 18 interaction P values are nominal and unadjusted for multiple comparisons. A significant result within one subgroup and a non-significant result within another do not establish an interaction.

## Table S14 Subgroup analyses and formal tests of interaction

| **Factor** | **Outcome** | **Subgroup (k) and effect estimate (95% CI)** | **P for subgroup differences** | **Outside factor comparison** |
| --- | --- | --- | --- | --- |
| Procedure type | 6MWD (MD, m) | Surgery (13): 51.59 (36.14 to 67.03) TAVR/TAVI (6): 10.20 (-25.28 to 45.68) | 0.036 | 0 |
| Procedure type | Peak VO₂ (SMD) | Surgery (3): 0.37 (0.12 to 0.63) TAVR/TAVI (5): 0.30 (-0.15 to 0.74) | 0.768 | 0 |
| Procedure type | LVEF (MD, pp) | Surgery (9): 5.26 (2.76 to 7.77) TAVR/TAVI (5): 2.40 (-1.74 to 6.53) | 0.246 | 0 |
| Procedure type | Hospital stay (MD, days) | Surgery (13): -1.80 (-2.53 to -1.06) / TAVR/TAVI (2): -2.25 (-4.07 to -0.43) | 0.649* | 1 (Mixed) |
| Procedure type | Adverse events (RR) | Surgery (12/13): 0.46 (0.26 to 0.82) TAVR/TAVI (2/6): 0.77 (0.32 to 1.84) | 0.335* | 0 |
| Procedure type | Pulmonary complications (RR) | Surgery (4): 0.63 (0.46 to 0.88) / TAVR/TAVI (2): 0.31 (0.11 to 0.93) | 0.220* | 1 (Mixed) |
| Intervention duration | 6MWD (MD, m) | ≤8 weeks (8): 31.12 (2.67 to 59.57) >8 weeks (4): 34.26 (24.65 to 43.88) | 0.837 | 7 |
| Intervention duration | Peak VO₂ (SMD) | ≤8 weeks (2): 0.40 (0.07 to 0.73) >8 weeks (4): 0.47 (0.22 to 0.72) | 0.754* | 2 |
| Intervention duration | LVEF (MD, pp) | ≤8 weeks (5): 5.42 (2.59 to 8.25) >8 weeks (3): 2.31 (-1.74 to 6.36) | 0.218 | 6 |
| Intervention duration | Hospital stay (MD, days) | ≤8 weeks (6): -0.48 (-0.89 to -0.07) >8 weeks (1): -8.40 (-10.74 to -6.06) | <0.001* | 9 |
| Intervention duration | Adverse events (RR) | ≤8 weeks (5/8): 1.03 (0.58 to 1.83) >8 weeks (3/4): 0.69 (0.12 to 3.80) | 0.661 | 7 |
| Intervention duration | Pulmonary complications (RR) | ≤8 weeks (3): 0.66 (0.52 to 0.83) >8 weeks (1): 0.18 (0.02 to 1.47) | 0.229* | 3 |
| Rehabilitation modality | 6MWD (MD, m) | AER (3): 48.68 (7.90 to 89.47) COMB (6): 27.84 (-12.20 to 67.87) RESP (3): 35.44 (-19.50 to 90.37) MULTI (7): 49.45 (27.88 to 71.03) | 0.798 | 0 |
| Rehabilitation modality | Peak VO₂ (SMD) | AER (3): 0.60 (0.26 to 0.95) COMB (3): 0.38 (0.08 to 0.69) RESP (1): -0.27 (-0.68 to 0.13) MULTI (1): 0.31 (-0.06 to 0.68) | 0.011* | 0 |
| Rehabilitation modality | LVEF (MD, pp) | AER (2): 0.31 (-7.14 to 7.77) COMB (3): 6.56 (5.14 to 7.98) RESP (2): 1.00 (-1.76 to 3.77) MULTI (7): 5.91 (2.67 to 9.16) | 0.003* | 0 |
| Rehabilitation modality | Hospital stay (MD, days) | AER (1): -3.26 (-5.72 to -0.80) COMB (2): -0.21 (-0.49 to 0.07) RESP (3): -1.22 (-1.93 to -0.50) MULTI (10): -2.36 (-3.36 to -1.35) | <0.001* | 0 |
| Rehabilitation modality | Adverse events (RR) | AER (0/1): Not estimable COMB (1/4): 0.67 (0.12 to 3.57) RESP (2/3): 0.61 (0.20 to 1.87) MULTI (11): 0.48 (0.26 to 0.90) | 0.896* | 0 |
| Rehabilitation modality | Pulmonary complications (RR) | RESP (1): 0.70 (0.51 to 0.96) MULTI (6): 0.56 (0.41 to 0.78) | 0.329* | 0 |

k = estimable trials; estimable/entered is shown when different. * At least one estimable subgroup contains fewer than three trials; these interaction tests are exploratory and do not support a firm subgroup claim. AER, aerobic training; COMB, combined exercise; RESP, respiratory training/IMT; MULTI, early mobilisation or multimodal rehabilitation. For AE modality, AER is not estimable; the test compares the other three categories. CI, confidence interval; LVEF, left ventricular ejection fraction; 6MWD, six-minute walk distance; peak VO₂, peak oxygen uptake. Mixed denotes a trial with both surgical and TAVR participants whose aggregate estimate is excluded only from the procedure comparison. MD, mean difference; SMD, standardized mean difference; RR, risk ratio; pp, percentage points; AE, adverse event; IMT, inspiratory muscle training; TAVR/TAVI, transcatheter aortic valve replacement/implantation; I², percentage of variability attributable to heterogeneity.

## Table S15 Sensitivity analyses

| **Outcome** | **Main random effects (95% CI)** | **Fixed effect (95% CI)** | **Exclude H-rated results (95% CI)** | **Leave-one-out estimate range** | **Timing restriction (95% CI)** | **Exclude Gao 2025 (95% CI)** |
| --- | --- | --- | --- | --- | --- | --- |
| 6MWD (MD, m) | 40.95 (27.10 to 54.81) | 39.99 (36.46 to 43.52) | 43.09 (29.00 to 57.17) P <0.001 k = 15; H removed: 4 | 38.02 to 46.23 Maximum P <0.001 | ≤30 d (k = 3): 44.80 (13.22 to 76.37) P = 0.005 >90 d (k = 6): 13.97 (-26.42 to 54.36) P = 0.498 | 42.14 (26.94 to 57.34) / P <0.001 / k = 18 |
| Peak VO₂ (SMD) | 0.32 (0.07 to 0.57) | 0.30 (0.12 to 0.47) | 0.33 (-0.14 to 0.80) P = 0.170 k = 5; H removed: 3 | 0.23 to 0.43 Maximum P = 0.055 | >90 d (k = 7): 0.23 (0.01 to 0.46) P = 0.043 | Not applicable; Gao 2025 does not contribute. |
| LVEF (MD, pp) | 4.41 (2.26 to 6.56) | 5.77 (5.13 to 6.41) | No H-rated result All 14 retained | 3.92 to 5.00 Maximum P <0.001 | >90 d (k = 3): -1.34 (-4.84 to 2.16) P = 0.453 | Not applicable; Gao 2025 does not contribute. |
| Hospital stay (MD, days) | -1.77 (-2.41 to -1.13) | -0.77 (-0.94 to -0.61) | -1.54 (-2.16 to -0.93) P <0.001 k = 13; H removed: 3 | -1.95 to -1.46 Maximum P <0.001 | Not applicable¹ | -1.79 (-2.47 to -1.11) / P <0.001 / k = 15 |
| Adverse events (RR) | 0.53 (0.33 to 0.85) | 0.53 (0.39 to 0.72) | 0.48 (0.27 to 0.85) P = 0.012 k = 11; H removed: 4 | 0.47 to 0.56 Maximum P = 0.024 | Not applicable¹ | 0.56 (0.33 to 0.92) / P = 0.023 / k = 13 |
| Pulmonary complications (RR) | 0.62 (0.50 to 0.77) | 0.61 (0.51 to 0.73) | 0.55 (0.36 to 0.85) P = 0.006 k = 4; H removed: 3 | 0.54 to 0.65 Maximum P = 0.002 | Not applicable¹ | 0.65 (0.55 to 0.77) / P <0.001 / k = 6 |

H, high risk; k, estimable trials. Only outcome-specific H results are excluded. No LVEF result is rated H, so all 14 remain. H exclusions: 6MWD—Rogers 2018, Vitez 2023, Sibilitz 2016, Ahmad 2024; peak VO₂—Tamulevičiūtė-Prascienė 2021, Vitez 2023, Sibilitz 2016; hospital stay—Lin 2004, Liu 2025, Ahmad 2024; adverse events—Sire 1987, Lin 2004, Vitez 2023, Shi 2025; PPC—Lin 2004, Shi 2025, Liu 2025. CI, confidence interval; LVEF, left ventricular ejection fraction; 6MWD, six-minute walk distance; peak VO₂, peak oxygen uptake. MD, mean difference; SMD, standardized mean difference; RR, risk ratio; pp, percentage points; PPC, postoperative pulmonary complication; TAVR, transcatheter aortic valve replacement; I², percentage of variability attributable to heterogeneity.

¹ Timing restrictions apply only to the selected functional outcomes. Windows were ≤30, 31–90, and >90 days after the valve procedure, with one month treated as 30 days. No selected result could be assigned unambiguously to 31–90 days. Results with an unclear anchor, a range crossing a boundary, or discharge without a reported postoperative day were omitted only from this restriction. A window was pooled when at least three studies remained; this threshold was not applied to every subgroup analysis. These revision-stage analyses retained the originally selected endpoint values and do not test differences between time windows. The >90-day LVEF analysis contains only TAVR studies. Omitting Tamulevičiūtė-Prascienė 2021 gave the maximum leave-one-out P value for peak VO₂ (0.055). The Gao 2025 exclusion examines uncertainty about the unit of randomisation without altering the principal dataset.

# Appendix 12 Funnel plots

Funnel plots were inspected visually for possible small-study effects. Asymmetry can also arise from heterogeneity and does not establish publication bias. Interpretation is particularly limited for peak VO₂ and pulmonary complications, each with fewer than ten studies. Five double-zero adverse-event trials do not provide an estimable RR and are not represented in its funnel plot. Outcome-specific judgments are reported in Table S16.

**Figure S1 6MWD (k = 19)	Figure S2 Peak VO₂ (k = 8)**

**Figure S3 LVEF (k = 14)	Figure S4 Hospital stay (k = 16)**

**Figure S5 Adverse events (k = 14)	Figure S6 Pulmonary complications (k = 7)**

# Appendix 13 GRADE evidence profile

## Table S16 GRADE evidence profile

| **Outcome** | **Trials entered / estimable** | **Effect estimate 95% CI** | **Risk of bias** | **Inconsistency** | **Indirectness** | **Imprecision** | **Publication bias** | **Certainty** |
| --- | --- | --- | --- | --- | --- | --- | --- | --- |
| 6MWD | 19 / 19 N = 1574 | MD, m 40.95 (27.10 to 54.81) | Serious (−1) | Serious (−1) | Serious (−1) | Not serious (0) | Uncertain (0) | Very low |
| Peak VO₂ | 8 / 8 N = 518 | SMD 0.32 (0.07 to 0.57) | Serious (−1) | Not serious (0) | Serious (−1) | Serious (−1) | Uncertain (0) | Very low |
| LVEF | 14 / 14 N = 1197 | MD, pp 4.41 (2.26 to 6.56) | Serious (−1) | Serious (−1) | Serious (−1) | Not serious (0) | Uncertain (0) | Very low |
| Hospital stay | 16 / 16 N = 2327 | MD, days -1.77 (-2.41 to -1.13) | Serious (−1) | Serious (−1) | Serious (−1) | Not serious (0) | Suspected (−1) | Very low |
| Adverse events | 19 / 14 N = 1438 | RR 0.53 (0.33 to 0.85) | Serious (−1) | Not serious (0) | Very serious (−2) | Serious (−1) | Uncertain (0) | Very low |
| Pulmonary complications | 7 / 7 N = 1554 | RR 0.62 (0.50 to 0.77) | Serious (−1) | Not serious (0) | Serious (−1) | Not serious (0) | Uncertain (0) | Low |

MD, mean difference; SMD, standardized mean difference; pp, percentage points; RR, risk ratio. Certainty is bounded at very low even when more than three downgrade levels are assigned. N denotes all participants entered for that outcome; the adverse-event RR is estimable in 14 trials with 1276 participants, while all 19 trials contain 1438 participants. Five double-zero trials remain in the dataset but do not estimate an RR. CI, confidence interval; LVEF, left ventricular ejection fraction; 6MWD, six-minute walk distance; peak VO₂, peak oxygen uptake. GRADE, Grading of Recommendations Assessment, Development and Evaluation; RoB, risk of bias.

# Reasons for the GRADE judgments

6MWD. Risk of bias was downgraded for four high-risk results (17.0% of weight) plus limitations in the remaining results. Inconsistency was serious (I² 90.1%; prediction interval −16.23 to 98.13 m). Programme and control differences and unclear timing in 10/19 results limit applicability to a defined pathway. The pooled CI did not warrant another precision downgrade. Possible funnel asymmetry was inconclusive amid marked heterogeneity; publication bias remains possible.

Peak VO₂. Three high-risk results contribute 43.7% of weight; their exclusion gives SMD 0.33 (−0.14 to 0.80). Differences in programme content, comparators and CPET context limit directness. SMD 0.07 to 0.57 spans a near-trivial to materially larger effect, warranting imprecision downgrading. I² 46.2% and overlapping study intervals did not justify another inconsistency downgrade. Eight studies cannot reliably establish or exclude publication bias.

LVEF. All 14 results have some concerns, including randomisation in 13, measurement in 10 and reporting in all 14; these cumulative limitations warrant one RoB level. Inconsistency was serious (I² 89.5%; prediction interval −3.89 to 12.71 pp). Timing is unclear in 10/14 results and clinical pathways differ. The pooled CI did not warrant an extra precision downgrade. Funnel irregularity is not an unambiguous small-study benefit pattern, so publication bias remains uncertain without a further deduction.

Hospital stay. Three high-risk results contribute 16.2% of weight. I² 90.8% and a prediction interval of −4.24 to 0.69 days indicate important inconsistency. Additional recovery measures and different discharge pathways limit attribution to exercise and applicability. The pooled CI is sufficiently precise for the average direction. Less precise studies tend to report larger reductions, supporting a suspected publication-bias downgrade; heterogeneity is an alternative explanation and the plot is not proof.

Adverse events. Four entered results are high risk; estimable high-risk results contribute 26.5% of weight. Two indirectness levels reflect incompatible event constructs and observation periods, mixing exercise-associated harms with general morbidity. Imprecision was downgraded for only 149 recorded events and a wide range of relative benefit. No extra inconsistency deduction was applied for the same event-definition problem. The 14-study plot is inconclusive; five double-zero trials do not prove absence of harms.

Pulmonary complications. Three high-risk results contribute 41.6% of weight. Different diagnostic composites, observation periods and prevention packages warrant one indirectness level. I² 17.9%, 382 events and RR 0.50 to 0.77 did not warrant further inconsistency or imprecision downgrading. Excluding high-risk results retains a protective estimate, RR 0.55 (0.36 to 0.85). Seven studies leave publication bias uncertain; small study count alone was not penalized.
